# FXYD3 functionally demarcates an ancestral breast cancer stem cell subpopulation with features of drug-tolerant persisters

**DOI:** 10.1172/JCI166666

**Published:** 2023-11-15

**Authors:** Mengjiao Li, Tatsunori Nishimura, Yasuto Takeuchi, Tsunaki Hongu, Yuming Wang, Daisuke Shiokawa, Kang Wang, Haruka Hirose, Asako Sasahara, Masao Yano, Satoko Ishikawa, Masafumi Inokuchi, Tetsuo Ota, Masahiko Tanabe, Kei-ichiro Tada, Tetsu Akiyama, Xi Cheng, Chia-Chi Liu, Toshinari Yamashita, Sumio Sugano, Yutaro Uchida, Tomoki Chiba, Hiroshi Asahara, Masahiro Nakagawa, Shinya Sato, Yohei Miyagi, Teppei Shimamura, Luis Augusto E. Nagai, Akinori Kanai, Manami Katoh, Seitaro Nomura, Ryuichiro Nakato, Yutaka Suzuki, Arinobu Tojo, Dominic C. Voon, Seishi Ogawa, Koji Okamoto, Theodoros Foukakis, Noriko Gotoh

**Affiliations:** 1 Division of Cancer Cell Biology, Cancer Research Institute, and; 2 Institute for Frontier Science Initiative, Kanazawa University, Kanazawa City, Japan.; 3 Division of Cancer Differentiation, National Cancer Center Research Institute, Chuo-ku, Tokyo, Japan.; 4 Department of Oncology-Pathology, Karolinska Institute, Karolinska University Hospital, Stockholm, Sweden.; 5 Division of Systems Biology, Graduate School of Medicine, Nagoya University, Nagoya City, Japan.; 6 Department of Breast and Endocrine Surgery, Graduate School of Medicine, The University of Tokyo, Bunkyo-ku, Tokyo, Japan.; 7 Department of Surgery, Minami-machida Hospital, Machida City, Tokyo, Japan.; 8 Department of Breast Oncology, Kanazawa University Hospital, Kanazawa City, Japan.; 9 Department of Breast and Endocrine Surgery, Nihon University, Itabashi-ku, Tokyo, Japan.; 10 Laboratory of Molecular and Genetic Information, Institute for Quantitative Biosciences, The University of Tokyo, Bunkyo-ku, Tokyo, Japan.; 11 Department of Oncology, Shanghai Medical College, Fudan University, Shanghai, China.; 12 North Shore Heart Research Group, Kolling Institute, University of Sydney, Sydney, New South Wales, Australia.; 13 Department of Breast and Endocrine Surgery, Kanagawa Cancer Center, Yokohama City, Kanagawa, Japan.; 14 Tokyo Medical and Dental University, Bunkyo-ku, Tokyo, Japan.; 15 Department of Systems Biomedicine, Tokyo Medical and Dental University, Bunkyo-ku, Tokyo, Japan.; 16 Department of Pathology and Tumor Biology, Graduate School of Medicine, Kyoto University, Sakyo-ku, Kyoto, Japan.; 17 Molecular Pathology and Genetics Division, Kanagawa Cancer Center Research Institute, Yokohama City, Kanagawa, Japan.; 18 Laboratory of Computational Genomics, Institute for Quantitative Biosciences,; 19 Department of Computational Biology and Medical Sciences, Graduate School of Frontier Biosciences,; 20 Department of Cardiovascular Medicine, Graduate School of Medicine,; 21 Genome Science Division, Research Center for Advanced Science and Technology,; 22 Department of Frontier Cardiovascular Science, Graduate School of Medicine, and; 23 Institute of Medical Science, The University of Tokyo, Tokyo, Japan.; 24 Inflammation and Epithelial Plasticity Unit, Cancer Research Institute, Kanazawa University, Kanazawa City, Japan.; 25 Advanced Comprehensive Research Organization, Teikyo University, Itabashi-ku, Tokyo, Japan.

**Keywords:** Oncology, Stem cells, Breast cancer, Drug therapy

## Abstract

The heterogeneity of cancer stem cells (CSCs) within tumors presents a challenge in therapeutic targeting. To decipher the cellular plasticity that fuels phenotypic heterogeneity, we undertook single-cell transcriptomics analysis in triple-negative breast cancer (TNBC) to identify subpopulations in CSCs. We found a subpopulation of CSCs with ancestral features that is marked by FXYD domain–containing ion transport regulator 3 (FXYD3), a component of the Na^+^/K^+^ pump. Accordingly, FXYD3^+^ CSCs evolve and proliferate, while displaying traits of alveolar progenitors that are normally induced during pregnancy. Clinically, FXYD3^+^ CSCs were persistent during neoadjuvant chemotherapy, hence linking them to drug-tolerant persisters (DTPs) and identifying them as crucial therapeutic targets. Importantly, FXYD3^+^ CSCs were sensitive to senolytic Na^+^/K^+^ pump inhibitors, such as cardiac glycosides. Together, our data indicate that FXYD3^+^ CSCs with ancestral features are drivers of plasticity and chemoresistance in TNBC. Targeting the Na^+^/K^+^ pump could be an effective strategy to eliminate CSCs with ancestral and DTP features that could improve TNBC prognosis.

## Graphical abstract

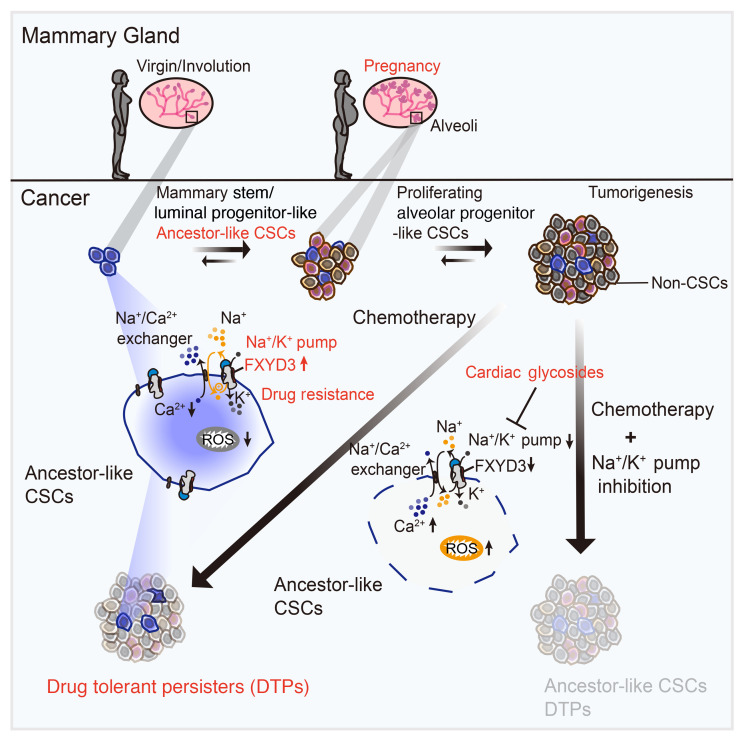

## Introduction

Patients with triple-negative breast cancer (TNBC), clinically defined by their minimal expression or absence of estrogen receptor, progesterone receptor, and receptor tyrosine-protein kinase erbB-2 (HER2), have poor prognosis due to the lack of effective molecular targeting therapies (1, 2). Cancer tissues comprise a heterogeneous population of cancer cells, including a small fraction with stemness traits, called cancer stem cells (CSCs) (3), which are believed to comprise drug-resistant subpopulations that can contribute to relapse. Previous studies have reported that the expression of various plasma membrane proteins, such as CD24^lo^CD44^hi^, CD133^+^, neuropilin-1 (NRP1), and insulin-like growth factor 1 receptor (IGF1R), or high aldehyde dehydrogenase (ALDH) activity can enrich for breast CSCs (1, 4–11). Each of these markers defines CSC subpopulations with overlapping yet distinct stem cell–like functions and targetable vulnerabilities while coexisting with other CSC-like cells. For example, IGF1R induces stemness through the transcription factor ID1 (9), whereas NRP1 induces symmetric division of CSCs (11). However, targeting CSCs presents a challenge due to the intratumoral heterogeneity. Moreover, the lack of definitive markers that can identify the drug-resistant subpopulation also hampers detailed understanding of the hierarchy within the CSC populations. It is also unclear whether CSC subgroups within tumors derive their traits from a common ancestral subpopulation.

Neoadjuvant chemotherapy (NAC) has been widely accepted as the standard of care before surgery for TNBC. This allows de-escalation in breast cancer surgery and reduces the risk of recurrence (2). However, where NAC failure causes a pathologically incomplete response in patients (12), there is a significantly greater risk of recurrence and worse survival. Several lines of recent evidence suggest that persistent residual tumor cells, often referred to as drug-tolerant persisters (DTPs), appear through a reversible adaptive response to chemotherapy (13–17). Although they have common clinical characteristics, the relationship between CSCs and DTPs awaits clarification.

Mammary glands develop during puberty, forming a network of ductal structures with 2 layers: an inner luminal cell layer and an outer myoepithelial cell layer (18, 19). A small number of mammary stem cells reside in the myoepithelial cell layer and differentiate into luminal progenitors that reside in the luminal cell layer, giving rise to all luminal cells. The onset of pregnancy initiates a highly proliferative phase of mammary development that is characterized by further ductal side branching and the development of widespread alveoli. During this process, luminal progenitors induce proliferative alveolar progenitors that, in turn, give rise to milk-producing differentiated alveoli. Recent single-cell transcriptomic profiling of mammary tissues at different developmental stages has elucidated the distinct genetic programs that define each of these cell populations (20).

The Na^+^/K^+^ ATPase pump is made up of α (ATP1A), β (ATP1B), and FXYD subunits. It maintains a resting plasma membrane potential by keeping higher K^+^ and lower Na^+^ concentrations in the cytoplasm than in the extracellular space (21). The ATP1A subunit possesses ATPase activity and powers the import of two K^+^ ions against the export of three Na^+^ ions. The FXYD subunit has several functions, one of which is to suppress reactive oxygen species–induced (ROS-induced) glutathionylation of specific cysteine (Cys) residues on ATP1B to maintain Na^+^/K^+^ pump activity (22, 23). FXYD domain–containing ion transport regulator 3 (FXYD3) has 2 isoforms — shorter FXYD3a and longer FXYD3b — that can differently regulate the affinity of the ATP1A subunit for Na^+^ and/or K^+^ (24–26). Cardiac glycosides have traditionally been used to treat heart failure by inhibiting the ATPase activity of the Na^+^/K^+^ pump and improving cardiomyocyte contractility (27). More recently, they have been repositioned as so-called senolytic drugs, which promote apoptosis and removal of senescent cells (28, 29).

To assess the heterogeneity within the CSC fraction of TNBC, we profiled TNBC cells from pre- and mid- to post-NAC primary tumors and patient-derived xenograft (PDX) tumors, as well as patient-derived cancer cells, at the single-cell level. We found ancestor-like CSCs that are defined by increased expression of FXYD3 in combination with other CSC markers. These cells have ability to differentiate into non-CSCs, and in turn, small numbers of non-CSCs could dedifferentiate into the ancestor-like CSCs, showing plasticity. Furthermore, increased FXYD3 expression enables the Na^+^/K^+^ pump in the ancestor-like CSCs to resist ROS, enhancing tumor resilience. Importantly, many of these traits are shared with DTPs. DTPs do not encompass all general CSC attributes, but are enriched with those that characterize FXYD3^hi^ CSCs.

Our findings represent a substantial advancement in the concept of CSCs by providing a mechanistic basis to comprehend the contribution of CSCs to DTPs, and uncover effective therapeutic targets to improve TNBC prognosis. Specifically, they suggest repurposing therapeutics targeting the Na^+^/K^+^ pump for use in combination with regular chemotherapies such as NAC to eliminate ancestor-like CSCs, thereby improving treatment efficacy and preventing cancer recurrence.

## Results

### Tumor cells with mammary immature traits correlate with drug resistance.

**Figure 1:**
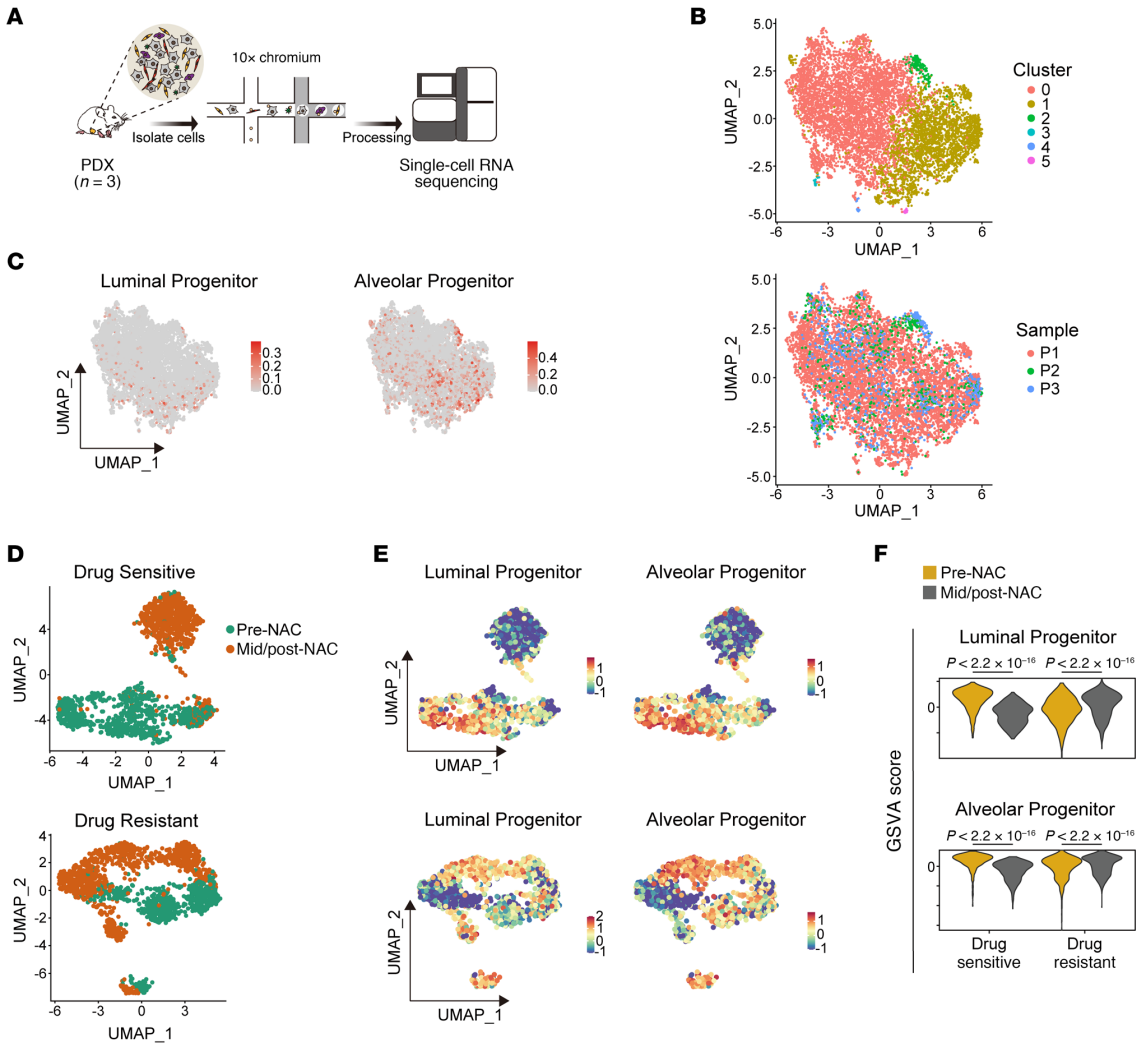
Tumor cells with mammary immature traits correlate with drug resistance. (**A**) Workflow of scRNA-Seq of patient-derived xenografts (PDXs). (**B**) UMAP visualization of scRNA-Seq data from 3 PDX samples (P1, P2, and P3), colored by their unsupervised clusters (top) and samples (bottom). (**C**) Gene set variation analysis (GSVA) score of gene signatures of luminal progenitors and alveolar progenitors. (**D**) UMAP visualization of integrated single-nucleus RNA-Seq profiles of 4 drug-sensitive and 4 drug-resistant patients, who received neoadjuvant chemotherapy (NAC). (**E** and **F**) GSVA score of gene signatures of mammary gland progenitors (**E**) compared between pre- and mid-/post-NAC subgroups (**F**). Wilcoxon’s rank sum test (**F**) was used to determine significant *P* values.

It is thought that breast cancer cells of origin are immature mammary epithelial cells, most likely luminal progenitor cells (30, 31). To interrogate whether CSCs with such ancestor-like traits exist in the tumor tissues, we profiled the transcriptome of three TNBC PDX models using single-cell RNA sequencing (scRNA-Seq) according to the droplet-based 10X Genomics protocol (Figure 1A and P1, P2, and P3 in Supplemental Table 1; supplemental material available online with this article; https://doi.org/10.1172/JCI166666DS1). While tumor epithelial cells were derived from humans, other cell types in the tumor microenvironment, such as immune cells, stromal cells, and endothelial cells, were mainly derived from mice, the xenograft host (32). We used uniform manifold approximation and projection (UMAP) on the scRNA-Seq data of 8,390 individual cells that were identified as human cells and found that these cells were resolved into several clusters without clear separations, as reported previously (33) (Figure 1B, top). The main clusters (clusters 0–2) comprised cells derived from all 3 samples (Figure 1B, bottom). The heatmap depicting the top 10 genes expressed in each cluster revealed distinct expression patterns that allowed for the differentiation of each cluster (Supplemental Figure 1A). Most of these cells were tumor epithelial cells, characterized by high expression of *keratin 19* (*KRT19*) and low expression of *PTPRC* (*CD45*), *PECAM1* (*CD31*), and *PDGFRB*, which are markers for immune/hematopoietic cells, endothelial cells, and stromal cells, respectively (34–36) (Supplemental Figure 1B). To interrogate whether mammary progenitor-like cells remain in cancer tissues, we analyzed the scRNA-Seq data for signature genes that represent immature mammary epithelial cells (20) (Supplemental Table 5). We observed cells expressing high levels of the signature genes of luminal progenitor cells and alveolar progenitor cells that appear only during pregnancy (Figure 1C), indicating that ancestor-like CSCs remain in cancer tissues. Additionally, tumor cells expressing elevated levels of the reported CSC markers *NRP1*, *IGF1R*, *CD44*, and *PROM1* (*CD133*) were scattered across several clusters (4, 7, 9, 11) (Supplemental Figure 1C). These results suggest limited heterogeneity in tumor cell populations.

We next analyzed single-nucleus RNA-Seq data of individual tumor cells from pre- and mid- to post-NAC TNBC tissues derived from the same patient (37). We performed integrated analysis of the snRNA-Seq data of pre-NAC tumor cells and mid- to post-NAC normal epithelial cells (i.e., the drug-sensitive and pathologically complete response group) derived from 4 NAC-sensitive cases. The cells derived from the pre-NAC and mid- to post-NAC samples were clearly separated into 2 clusters (Figure 1D, top). Likewise, similar integrated analysis using the snRNA-Seq data of 4 NAC-resistant cases showed clear separation into 2 clusters, one with pre-NAC tumor cells and the other with mid- to post-NAC tumor cells (Figure 1D, bottom). Although the expression levels of signature genes of luminal progenitor and alveolar progenitor cells were diminished after NAC in NAC-sensitive cancer tissues (in which only remaining normal epithelial cells were detectable), they were upregulated after NAC in chemotherapy-resistant cancer tissues (Figure 1, E and F). Analysis of bulk RNA-Seq data showed poor prognosis in patients with TNBC with high expression levels of luminal progenitor signature genes in their cancer tissues (38) (Supplemental Figure 1D). These results demonstrate the existence of tumor cells with traits of immature mammary epithelial cells and indicate that these cells contribute to poor prognosis and drug resistance in breast cancer.

### Traits of ancestor-like and other CSCs.

**Figure 2:**
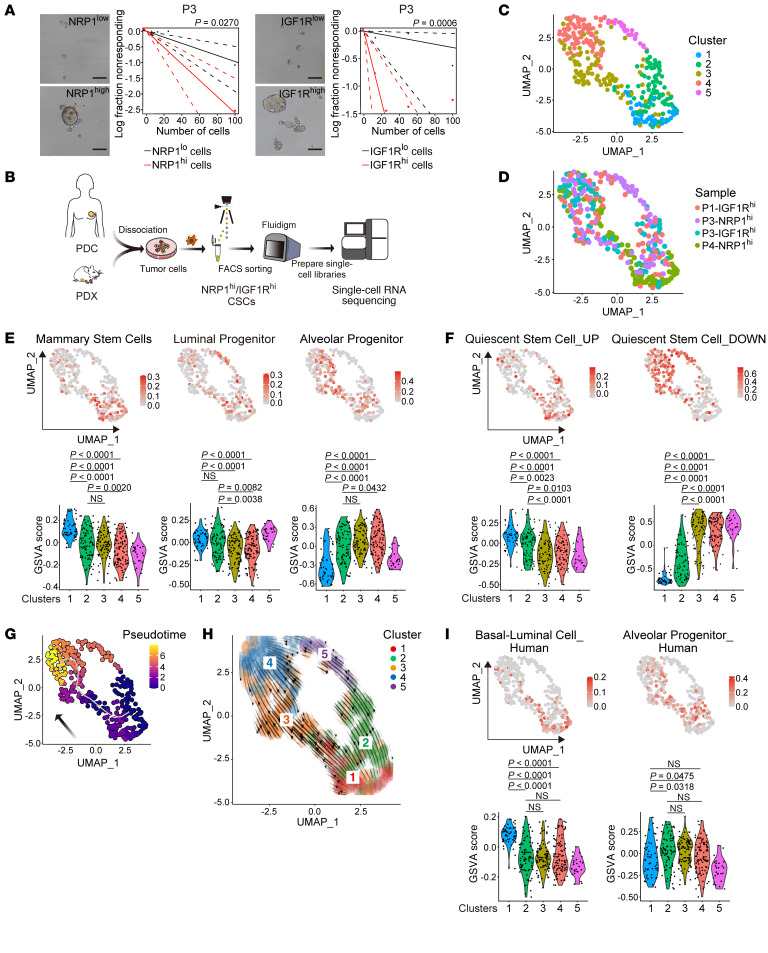
Ancestor-like CSCs possess mammary stem– or luminal progenitor–like traits and quiescence. (**A**) Tumor spheroids and data of the extreme limiting dilution assay (ELDA) of P3-derived cancer cells. Scale bars: 100 μm. (**B**) Graphical scheme describing the workflow of scRNA-Seq of breast CSCs. (**C** and **D**) UMAP visualization of SMART-seq data from all the cells in 4 cell populations (IGF1R^hi^ cells in P1, NRP1^hi^ cells in P3, IGF1R^hi^ cells in P3, and NRP1^hi^ cells in P4), colored by their unsupervised clusters (**C**) and samples (**D**). (**E**) Top: GSVA score of gene signatures of mammary gland stem/progenitors. Bottom: Violin plots of GSVA score for each cluster. (**F**) Top: GSVA score of quiescent stem cell gene signatures. Bottom: Violin plots of GSVA score for each cluster. (**G**) UMAP visualization of SMART-seq data from the cells colored by pseudotime. (**H**) UMAP visualization of RNA velocity derived from UniTVelo methods. (**I**) Top: GSVA score of gene signatures of human mammary gland immature cells. Bottom: Violin plots of GSVA score for each cluster. Statistical significance in **E**, **F**, and **I** was determined by 1-way ANOVA with Bonferroni’s post hoc test.

To further decipher the heterogeneity in CSCs, we used 2 plasma membrane markers, NRP1 and IGF1R, to enrich for CSCs (9, 11). Patient-derived cancer cells were sorted using anti-NRP1 or anti-IGF1R antibodies and cultured in floating spheroid-forming conditions for CSC enrichment (Supplemental Figure 2A, left). As expected, the extreme limiting dilution assay (ELDA) revealed that NRP1^hi^ or IGF1R^hi^ cells had significantly (*P* < 0.05 and *P* < 0.001, respectively) higher spheroid-forming ability than NRP1^lo^ or IGF1R^lo^ cells (Figure 2A and Supplemental Figure 2A, right). CSCs may comprise a minute proportion (1%–5%) of the total tumor epithelial cells in breast cancer tissues. Consistently, few cells with high expression of NRP1 or IGF1R could be found in the PDXs according to the UMAP results of our scRNA-Seq data (Supplemental Figure 1C). Next, we performed transcriptomic profiling of individual cells in NRP1^hi^ or IGF1R^hi^ CSC-enriched tumor cells derived from 2 treatment-naive patients (P1 and P3 in Supplemental Table 1) and tumor cells derived from 1 chemotherapy-resistant patient (P4), either from the PDX model (P1) or from primary cultures (P3 and P4; Figure 2B and Supplemental Figure 2B). We profiled NRP1^hi^ cells from P4 and P3 samples and IGF1R^hi^ cells in P1 and P3 samples. To detect subtle transcriptomic differences across a few hundred cells in a relatively homogenous CSC population, we used microfluidic technology (Fluidigm C1; https://www.crig.ugent.be/en/fluidigm-c1-single-cell-auto-prep-system) to conduct deep profiling of approximately 10,000 genes in individual cells (39, 40). Integrated analysis of scRNA-Seq data derived from all samples revealed 5 clusters, of which clusters 2–4 comprise all samples (Figure 2, C and D). The signature of luminal progenitors and their representative genes, *ALDH1A3* and *AREG*, was enriched in clusters 1 and 2, whereas the signature of alveolar progenitors and their representative genes, *CENPA* and *CDK1*, was enriched in clusters 3 and 4 (Figure 2E and Supplemental Figure 2C). We further analyzed the signature genes of the most immature cell type, the mammary stem cell (Supplemental Table 5), and found them to be enriched in cluster 1. The mammary stem cell markers *NOTCH3* and *BCAM* were expressed in some of the cells in cluster 1 (Supplemental Figure 2C). *BCL11B*, a recently reported intrinsic regulator of mammary stem cell quiescence, was specifically expressed in cluster 1 (41) (Supplemental Figure 2D). It is believed that many adult tissue-specific stem cells are quiescent or in G_0_ phase. Consistently, the quiescent stem cell gene signature (42) (Supplemental Table 5) was upregulated in clusters 1 and 2, and downregulated in clusters 3 and 4 (Figure 2F). The expression level of *MKI67*, which is a marker for proliferating cells (2), showed that cells in clusters 1 and 2 were not proliferating (Supplemental Figure 2E). Pseudotime analysis was used to infer the continuum of cell lineage development of clusters 1→2→3→4 (43), recapitulating normal mammary cell lineage development (Figure 2G). RNA velocity analysis showed similar direction of arrows, confirming cell lineage development (44) (Figure 2H). Since these signatures are derived from genes expressed in mouse mammary gland epithelial cells, we next analyzed recently reported human mammary gland single-cell data (45) (Supplemental Table 5). Human basal-luminal cell signature genes showed similar patterns to those of the mammary stem cell or luminal progenitor signature (Figure 2I). Additionally, human alveolar progenitor signature genes showed mixed patterns of the luminal and alveolar progenitor signatures. Therefore, the quiescent cells had the traits of the ancestral CSCs, hereafter, “ancestor-like CSCs.” Interestingly, the cells with traits of alveolar progenitor cells were the main component of proliferative CSCs.

Clustering of enriched Gene Ontology (GO) pathways in each cell clearly resolved them into 2 main groups: one of cells in clusters 1 and 2, and the other of cells in clusters 3 and 4. In cluster 1 and 2 cells, fatty acid metabolism and macroautophagy, the common characteristics of quiescent stem cells (46), were upregulated (Supplemental Figure 2, F and G). In contrast, in clusters 3 and 4, cell proliferation–related pathways were upregulated, such as positive regulation of the cell cycle process and DNA replication (Supplemental Figure 2, F and G). Cell cycle analysis revealed that most cells in clusters 1 and 2 were in G_0_ phase (Supplemental Figure 3, A and B).

In proliferating cells, functional genes that can independently regulate the cell cycle alone are expressed and might affect the data. Thus, we reanalyzed the RNA-Seq data after removing the effects of such genes. The results showed 3 clusters, each comprising cells derived from all the samples (Supplemental Figure 4A). The expression level of *MKI67* showed that cells in cluster 1 were not proliferating (Supplemental Figure 4B). Mammary stem cell signature was enriched in cluster 1, luminal progenitor signature was enriched in clusters 1 and 2, and alveolar progenitor signature was enriched in cluster 3 (Supplemental Figure 4C). Pseudotime analysis showed cell lineage development of clusters 1→2→3 (Supplemental Figure 4D). All these results corroborate our findings, indicating that the quiescent immature cells are the ancestor-like CSCs.

### FXYD3 expression demarcates ancestor-like CSCs.

**Figure 3:**
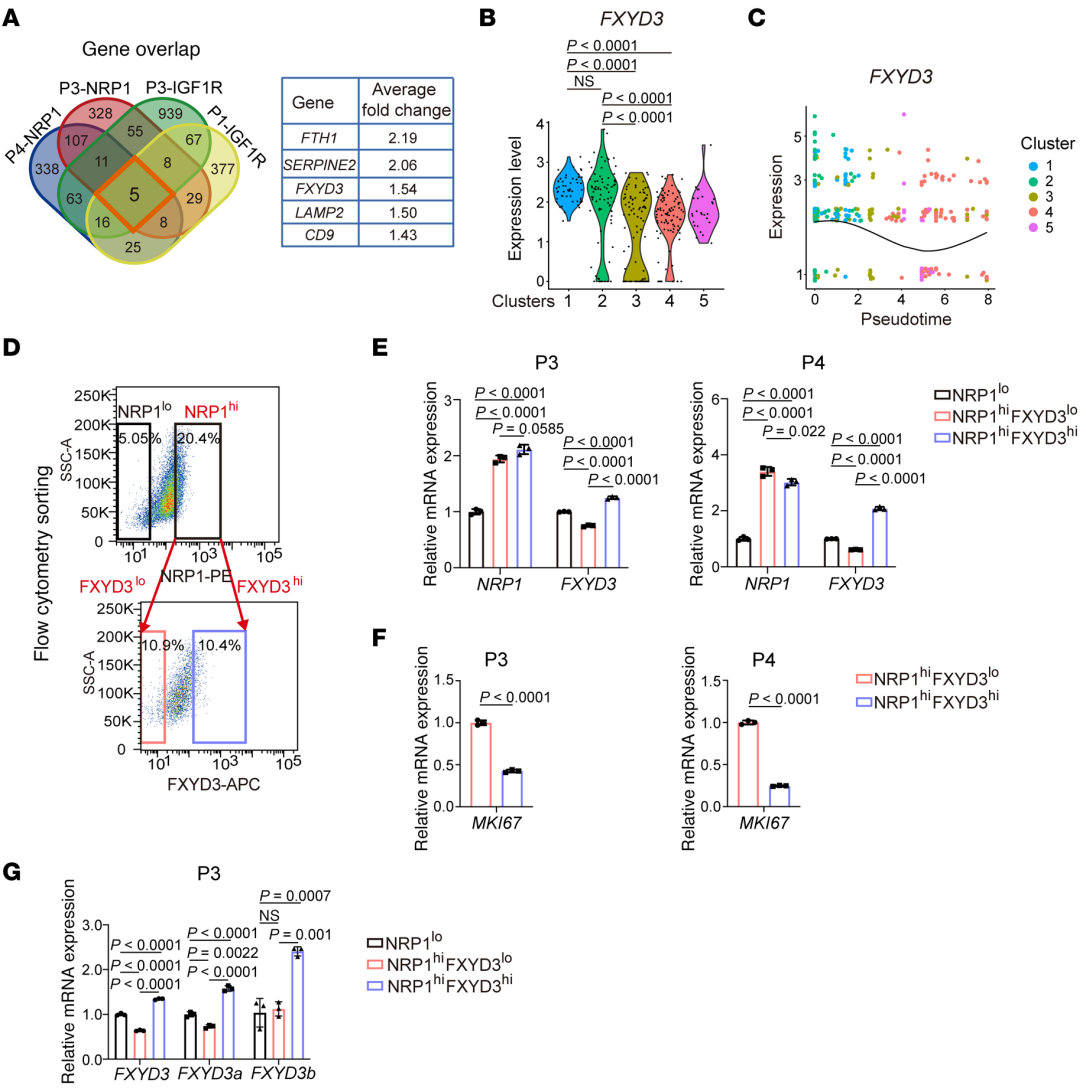
Plasma membrane FXYD3 demarcates ancestor-like CSCs. (**A**) Venn diagram of upregulated genes (log_2_[fold change] > 0.2, Wilcoxon’s rank sum test *P* < 0.05) in the quiescent clusters (MKI67^lo^), compared with genes in other clusters of the SMART-Seq data. (**B**) Violin plots of FXYD3 expression (Seurat, https://satijalab.org/seurat/ log[normalized counts]) in each cluster shown in Figure 2C. Statistical significance was determined by Kruskal-Wallis test with Dunn’s multiple-comparison test. (**C**) Changes in FXYD3 expression (Seurat, log[normalized counts]) during pseudotime. (**D**) FACS sorting strategy using combination of NRP1 and FXYD3 antibodies. (**E**–**G**) Relative mRNA expression of NRP1, FXYD3, FXYD3a, FXYD3b, and MKI67 measured by quantitative PCR (qPCR) between NRP1^lo^, NRP1^hi^FXYD3^lo^, and NRP1^hi^FXYD3^hi^ cells. Values were normalized to ACTB, and fold changes were calculated relative to the values of NRP1^lo^ (**E** and **G**) or NRP1^hi^FXYD3^lo^ (**F**) cells. (**E** and **G**) Statistical significance was determined by 1-way ANOVA with Bonferroni’s post hoc test. (**F**) Statistical significance was determined by unpaired, 2-tailed Student’s *t* tests. Results are shown as means ± SD. *n* = 3.

To identify plasma membrane proteins that define ancestor-like CSCs, we analyzed scRNA-Seq data of individual samples. Cells from each sample were clearly resolved into MKI67^lo^ quiescent cell cluster and other proliferative cell clusters. Among the significantly upregulated genes in the quiescent cell cluster in comparison with other cell clusters, we obtained 5 commonly upregulated genes across all 4 samples (Figure 3A). We then focused on the plasma membrane protein FXYD3, as antibody-based cell sorting strategies targeting FXYD3 can distinguish the ancestor-like CSCs. In the integrated clusters derived from all 4 samples (Figure 2C), *FXYD3* was upregulated in clusters 1 and 2 compared with that in clusters 3 and 4 (Figure 3B). Concordantly, the expression level of *FXYD3* was highest in clusters 1 and 2, and gradually decreased during pseudotime-inferred cell lineage development (Figure 3C). Similar trends were observed in other genes (Supplemental Figure 5, A and B). Analysis of The Cancer Genome Atlas database showed that *FXYD3* was upregulated in breast cancer tissues and not in normal tissues (Supplemental Figure 5C), and breast cancer patients with high *FXYD3* expression showed poor prognosis (Supplemental Figure 5D).

Our findings suggest that FXYD3^hi^ cells among the NRP1^hi^ or IGF1R^hi^ cell populations define ancestor-like CSCs, whereas FXYD3^lo^ cells are proliferative alveolar progenitor–like CSCs. Cancer cells were sorted using anti-FXYD3 and anti-NRP1 or anti-IGF1R antibodies. Quantitative PCR corroborated that each subpopulation was appropriately enriched (Figure 3, D and E, and Supplemental Figure 5, E and F). The expression level of *MKI67* was lower in FXYD3^hi^ cells than in FXYD3^lo^ cells among the NRP1^hi^ or IGF1R^hi^ CSCs, reflecting the quiescent state of ancestor-like CSCs (Figure 3F and Supplemental Figure 5G). Both *FXYD3* isoforms were expressed in NRP1^hi^FXYD3^hi^ ancestor-like CSCs (Figure 3G), with *FXYD3a* showing higher expression levels compared with *FXYD3b* (Supplemental Figure 5H).

**Figure 4:**
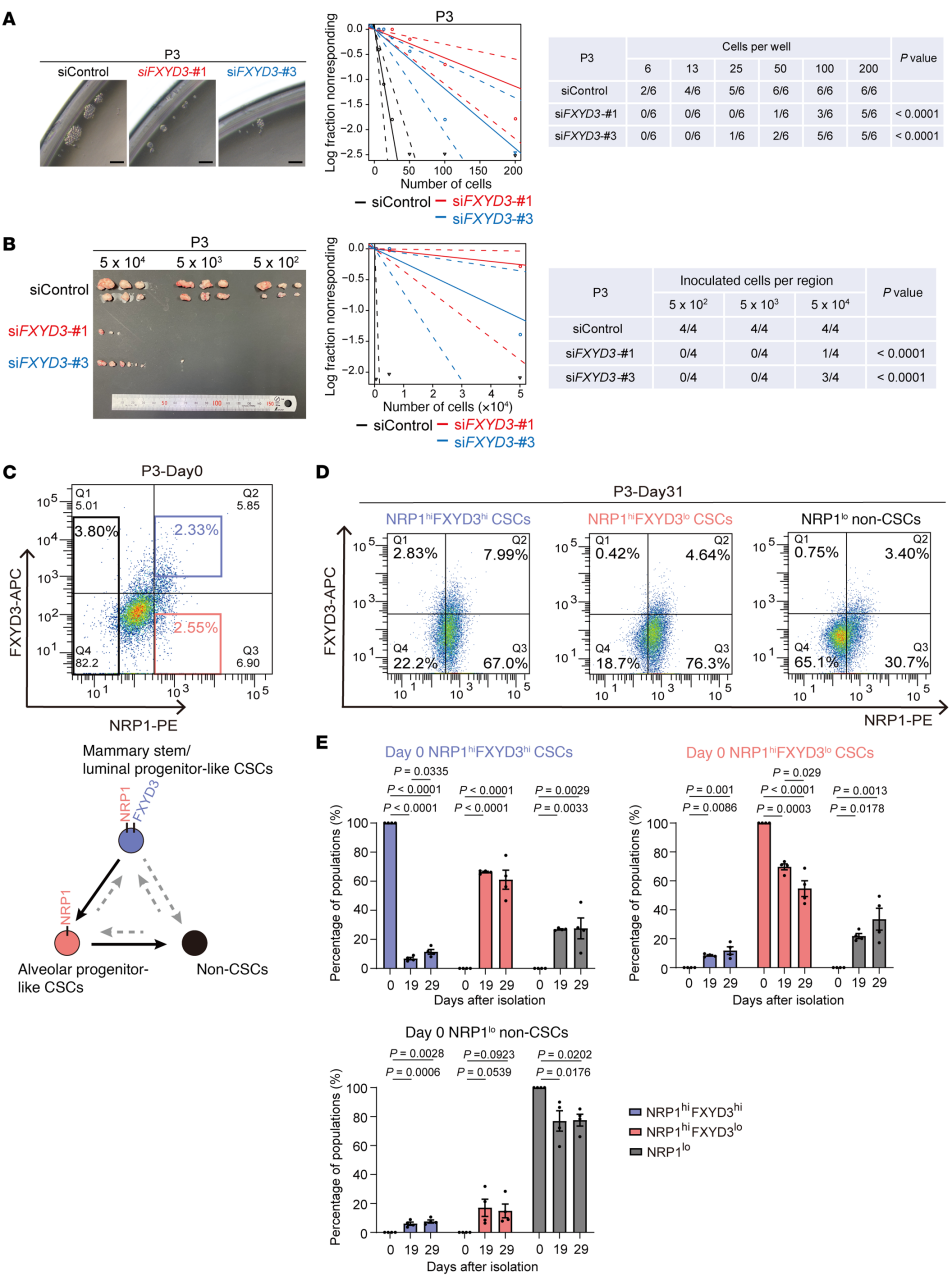
FXYD3 expression demarcates ancestor-like CSCs and cellular plasticity of each CSC population. (**A**) Tumor spheroids and data of the ELDA of P3-derived cancer cells and their FXYD3-knockdown cells in vitro. Scale bars: 50 μm. (**B**) Images of tumors generated in mice and data of the ELDA of P3-derived cancer cells and their FXYD3-knockdown cells in vivo. (**C**) FACS sorting (day 0) according to the expression levels of NRP1 and FXYD3. (**D**) FACS plot of cells in each population after culture for 31 days in the organoid medium. (**E**) Quantification of each population of NRP1^hi^FXYD3^hi^, NRP1^hi^FXYD3^lo^, and NRP1^lo^ cells after culture for 19 and 29 days in the organoid medium. Statistical significance was determined by 1-way ANOVA with Tukey’s multiple-comparison test. Results are shown as means ± SEM. *n* = 4.

To investigate the functional role of FXYD3 in enriching CSCs, we used siRNA to knock down both isoforms of *FXYD3* (Supplemental Figure 6A). ELDA analysis demonstrated that the knockdown of *FXYD3* remarkably decreased the spheroid-forming ability in vitro and tumor-initiating ability in vivo (Figure 4, A and B), providing strong evidence to support the notion that FXYD3 plays a crucial role in CSCs. Immunocytochemistry revealed that FXYD3 expression levels were higher in Ki67^–^ cells compared with Ki67^+^ cells within the NRP1^+^ cell population (Supplemental Figure 6B). Collectively, these results indicate that FXYD3^hi^ cells among the NRP1^hi^ or IGF1R^hi^ CSCs represent quiescent ancestor-like CSCs. Furthermore, we analyzed C1-based scRNA-Seq data obtained from NRP1- or IGF1R-sorted CSC fraction to examine the expression levels of *ALDH1A3* and *CD44* in FXYD3^hi^ ancestor-like CSCs. *ALDH1A3* or *CD44* did not exhibit high expression in half (51.6%) of the FXYD3^hi^ ancestor-like CSCs. Nevertheless, *CD44* showed elevated expression in about 40% (15.6% + 28.1%) of the FXYD3^hi^ ancestor-like CSCs, whereas a lesser proportion (~20%) (15.6% + 4.6%) of cells demonstrated high *ALDH1A3* expression. These findings indicate that *CD44* is expressed in a significant portion of the ancestor-like CSCs in an overlapping manner, suggesting a potential cooperative function between CD44 and FXYD3 in the ancestor-like CSC population.

### Cellular plasticity of each CSC population.

To explore whether CSCs undergo differentiation in vitro along with the inferred cell lineage, we prepared single-cell suspensions of each subpopulation, strictly gated to avoid cross-contamination, and cultured them in organoid media conducive to the maintenance of heterogeneous cell populations, including stem and differentiated cells (47, 48) (Figure 4C, top, and Supplemental Table 2). When NRP1^hi^FXYD3^hi^ ancestor-like CSCs were cultured for 19 and 29 or 31 days, most cells were NRP1^hi^FXYD3^lo^ alveolar progenitor–like CSCs, with a lesser number of NRP1^lo^ non-CSCs, indicating cell lineage development (Figure 4D, left, and Figure 4E, top left). Next, we cultured NRP1^hi^FXYD3^lo^ alveolar progenitor–like CSCs. Although some NRP1^lo^ non-CSCs appeared, a few NRP1^hi^FXYD3^hi^ ancestor-like CSCs were observed after 19 and 29 or 31 days (Figure 4D, middle, and Figure 4E, top right). Intriguingly, when NRP1^lo^ non-CSCs were cultured, a few NRP1^hi^FXYD3^hi^ ancestor-like CSCs appeared after 19 and 29 or 31 days (Figure 4D, right, and Figure 4E, bottom). Therefore, cancer cells may possess intrinsic cellular plasticity that enables them to dedifferentiate into ancestor-like CSCs (Figure 4C, bottom).

### FXYD3^hi^ ancestor-like CSCs develop drug resistance by harnessing Na^+^/K^+^ pump activity to reduce ROS production.

**Figure 5:**
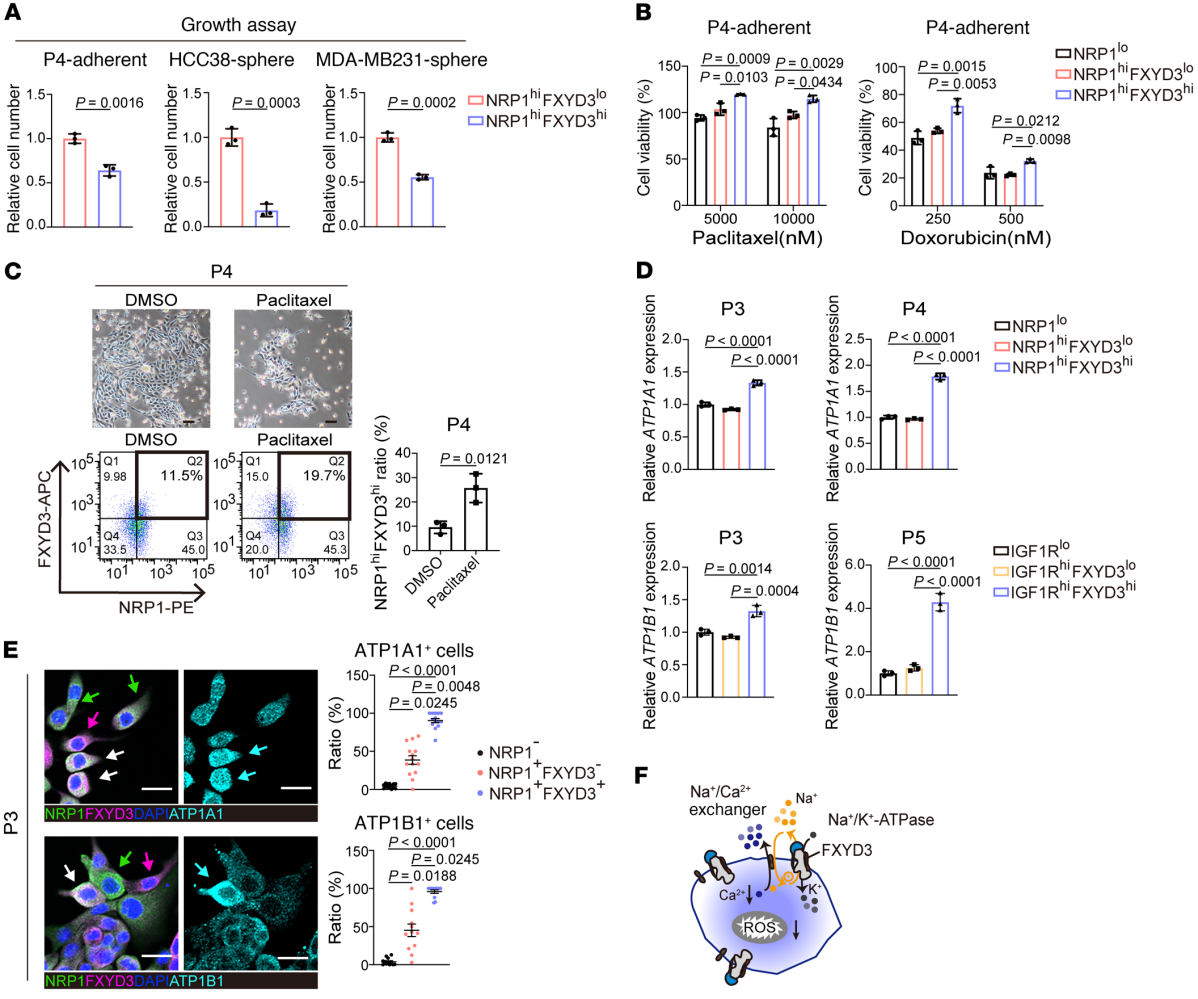
Drug resistance of FXYD3^hi^ ancestor-like CSCs by harnessing of the Na^+^/K^+^ pump. (**A** and **B**) Cell growth (**A**) and drug sensitivity assays (**B**). *n* = 3. (**C**) P4 patient-derived cancer cells after 48 hours of paclitaxel (10 μM) treatment. Scale bars: 100 μm. Bottom left: FACS analysis. Bottom right: The ratio (percent) of NRP1^hi^FXYD3^hi^ cells to total cells was quantitated based on FACS analysis. *n* = 3. (**D**) Relative mRNA expression levels of ATP1A1 and ATP1B1 measured by qPCR. Values were normalized to ACTB, and fold changes were calculated relative to the values of NRP1^lo^ cells. *n* = 3. (**E**) Left: Immunofluorescence staining of P3 cells using antibodies against NRP1, FXYD3, ATP1A1, or ATP1B1. Nuclei were stained by DAPI. Green arrows indicate cells positive for NRP1 but negative for FXYD3. Red arrows indicate cells negative for NRP1 but positive for FXYD3. White arrows indicate cells double-positive for NRP1 and FXYD3. Blue arrows indicate cells triple-positive for NRP1, FXYD3, and ATP1A1, or NRP1, FXYD3, and ATP1B1. Scale bars: 20 μm. Right: Ratio (percent) of ATP1A1-positive cells or ATP1B1-positive cells to total number of NRP1-negative cells, NRP1-positive but FXYD3-negative cells, or NRP1-positive and FXYD3-positive cells. Statistical significance was determined by Kruskal-Wallis test with Dunn’s multiple-comparison test. Results are shown as means ± SEM. *n* = 14 random fields for ATP1A1 and *n* = 12 random fields for ATP1B1 were counted. (**F**) Function of Na^+^/K^+^ pump. (**A** and **C**) Statistical significance was determined by unpaired, 2-tailed Student’s *t* tests. (**B** and **D**) Statistical significance was determined by 1-way ANOVA with Bonferroni’s post hoc test. Results are shown as means ± SD.

Cell proliferation analysis corroborates the lower proliferative activity in NRP1^hi^FXYD3^hi^ ancestor-like CSCs cultured in adherent and spheroid growth conditions (Figure 5A). To examine drug sensitivity, we treated cancer cells with the clinically used chemotherapeutic agents paclitaxel, doxorubicin, and olaparib, a poly(ADP-ribose) polymerase (PARP) inhibitor. Numerous NRP1^hi^FXYD3^hi^ ancestor-like CSCs survived and were enriched by the treatment with chemotherapeutic agents (Figure 5, B and C, and Supplemental Figure 7, A–D). Treatment with paclitaxel did not lead to an increase in the expression of FXYD3 in FXYD3^lo^ cells (Supplemental Figure 7E). Consequently, these findings indicate that ancestor-like CSCs are resistant to clinically used chemotherapeutic agents.

**Figure 6:**
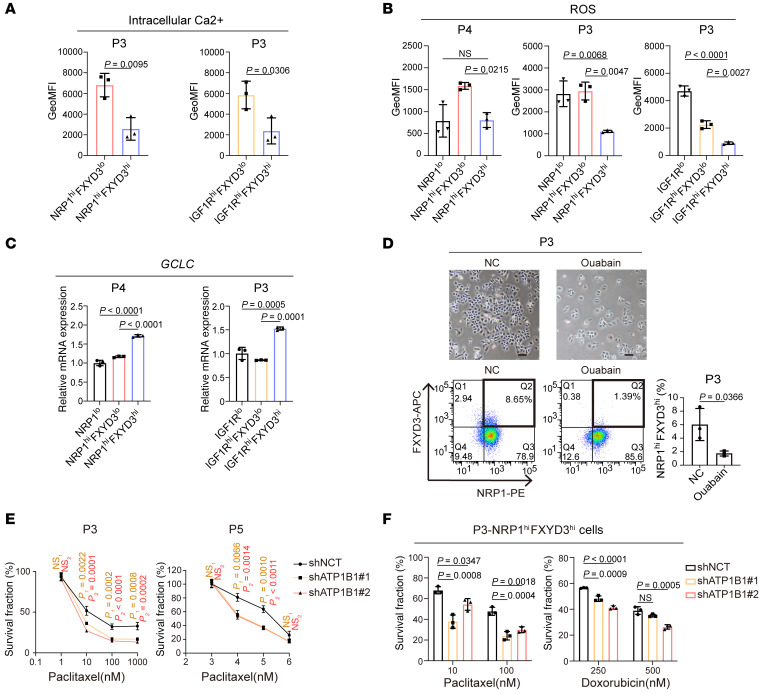
Na^+^/K^+^ pump inhibition decreases the proportion of FXYD3^hi^ ancestor-like CSCs and sensitizes them to drugs. (**A**) Geometric mean of fluorescence intensity (GeoMFI) of intracellular Ca^2+^ levels. *n* = 3. (**B**) GeoMFI of cellular ROS levels. *n* = 3. (**C**) Relative mRNA expression levels of GCLC measured by qPCR. Values were normalized to ACTB, and fold changes were calculated relative to the values of NRP1^lo^ cells. *n* = 3. (**D**) P3 cells after treatment with Na^+^/K^+^ pump inhibitor ouabain (50 nM) or vehicle alone (negative control [NC]). Scale bars: 100 μm. Bottom left: FACS analysis. Bottom right: The ratio (percent) of NRP1^hi^FXYD3^hi^ cells to total cells was quantitated based on FACS analysis. (**E**) After knockdown of ATP1B1, cells were treated with paclitaxel with serial concentrations for 72 hours. *n* = 3. (**F**) After knockdown of ATP1B1, NRP1^hi^FXYD3^hi^ cells were sorted by FACS and treated with paclitaxel or doxorubicin with serial concentrations for 72 hours. Statistical significance was determined by 1-way ANOVA with Dunnett’s multiple-comparison test. *n* = 3. (**A**, **D**, and **E**) Statistical significance was determined by unpaired, 2-tailed Student’s *t* tests. (**B** and **C**) Statistical significance was determined by 1-way ANOVA with Bonferroni’s post hoc test. Results are shown as means ± SD.

GO pathway analyses revealed that membrane potential–related pathways were upregulated in the cells of clusters 1 and 2, the ancestor-like CSCs (Supplemental Figure 2, F and G). Quantitative PCR analysis shows that the expression levels of *ATP1A1* and *ATP1B1*, encoding the α1 subunit and β1 subunit of the Na^+^/K^+^ pump, respectively, were higher in FXYD3^hi^ ancestor-like CSCs than in FXYD3^lo^ alveolar CSCs and non-CSCs (Figure 5D). Likewise, immunocytochemistry demonstrated that ATP1A1^hi^ or ATP1B1^hi^ cells were more abundant in the FXYD3^hi^ ancestor-like CSC population than in the FXYD3^lo^ alveolar CSC or NRP1^lo^ non-CSC populations (Figure 5E). These results indicate that Na^+^/K^+^ pump activity is upregulated in ancestor-like CSCs. The active Na^+^/K^+^ pump increases Na^+^ efflux from the cytoplasm, thereby activating the linked Na^+^/ Ca^2+^ exchanger (NCX) to increase cytoplasmic Na^+^ influx and Ca^2+^ efflux (49) (Figure 5F). This leads to a reduced intracellular concentration of Ca^2+^ ions, resulting in decreased ROS levels (50). Consistent with these findings, GO pathway analysis showed negative regulation of Ca^2+^-mediated signaling pathways in ancestor-like CSCs (Supplemental Figure 2, F and G). Indeed, the concentration of intracellular Ca^2+^ and ROS was significantly lower in FXYD3^hi^ ancestor-like CSCs than in FXYD3^lo^ CSCs (Figure 6, A and B). Furthermore, the activity of glutamate-Cys ligase catalytic (GCLC) subunit, a rate-limiting enzyme that catalyzes the production of the antioxidant glutathione (51), and redox status, expressed as the ratio of the reduced form of glutathione (GSH) to its oxidized form glutathione disulfide (GSSG), were increased, suggesting decreased ROS levels and a balanced redox status in FXYD3^hi^ ancestor-like CSCs (Figure 6C and Supplemental Figure 7F).

To examine whether ATPase is required for the maintenance of ancestor-like CSCs, we treated cells with cardiac glycosides, pharmacological inhibitors of ATPase in the Na^+^/K^+^ pump. Treatment with ouabain or digoxin at a half-maximal inhibitory concentration (IC_50_; Supplemental Figure 7G) significantly diminished the FXYD3^hi^ ancestor-like CSC population when compared with other cell populations, indicating that ancestor-like CSCs are dependent on Na^+^/K^+^ pump activity for their maintenance (Figure 6D and Supplemental Figure 7, H–J). Furthermore, we observed that ouabain showed a synergistic effect when administered in combination with paclitaxel on patient-derived cancer cells (Supplemental Figure 7K).

### ATP1B1 knockdown or treatment with ouabain sensitizes TNBC PDX tumors to paclitaxel treatment.

We found that knockdown of *FXYD3* led to a marked decrease in cancer cell proliferation, as reported previously (25, 52) (Supplemental Figure 8, A and B). This indicates that FXYD3 is essential for the proliferation of cancer cells and may play a critical role not only in the maintenance of ancestor-like CSCs but also in the proliferation of differentiated cancer cells. It has also been reported that knockdown of *FXYD3* leads to increased chemosensitivity (53).

We then performed shRNA-mediated knockdown of *ATP1B1* in patient-derived cancer cells (Supplemental Figure 8C). While in vitro cell proliferation was not significantly affected in *ATP1B1*-knockdown patient-derived P3 cells, it was mildly but significantly reduced in P5 cells (Supplemental Figure 8D). Sensitivity to paclitaxel or doxorubicin was markedly increased upon *ATP1B1* knockdown in bulk cells (Figure 6E) and in sorted NRP1^hi^FXYD3^hi^ ancestor-like CSCs (Figure 6F). The levels of *ATP1B3*, another member of the ATP1B family, were found to be higher in P3 cells than in P5 cells, whereas *ATP1B1* expression showed the inverse pattern (Supplemental Figure 8E). Notably, siRNA-mediated *ATP1B3* knockdown resulted in reduced proliferation in P3 cells (Supplemental Figure 8F), suggesting that P3 cells rely more on ATP1B3 than ATP1B1 for cell proliferation, likely because of the higher *ATP1B3* expression in P3 cells compared with P5 cells. Although ATP1B1 does not strongly contribute to proliferation of P3 cells, it appears to confer on them resistance to paclitaxel and doxorubicin. Furthermore, ROS production was significantly increased, whereas GCLC expression was substantially decreased, in ancestor-like CSCs upon *ATP1B1* knockdown (Supplemental Figure 8, G and H). These results indicate that *ATP1B1* knockdown–mediated decrease in Na^+^/K^+^ pump activity alleviates drug resistance.

**Figure 7:**
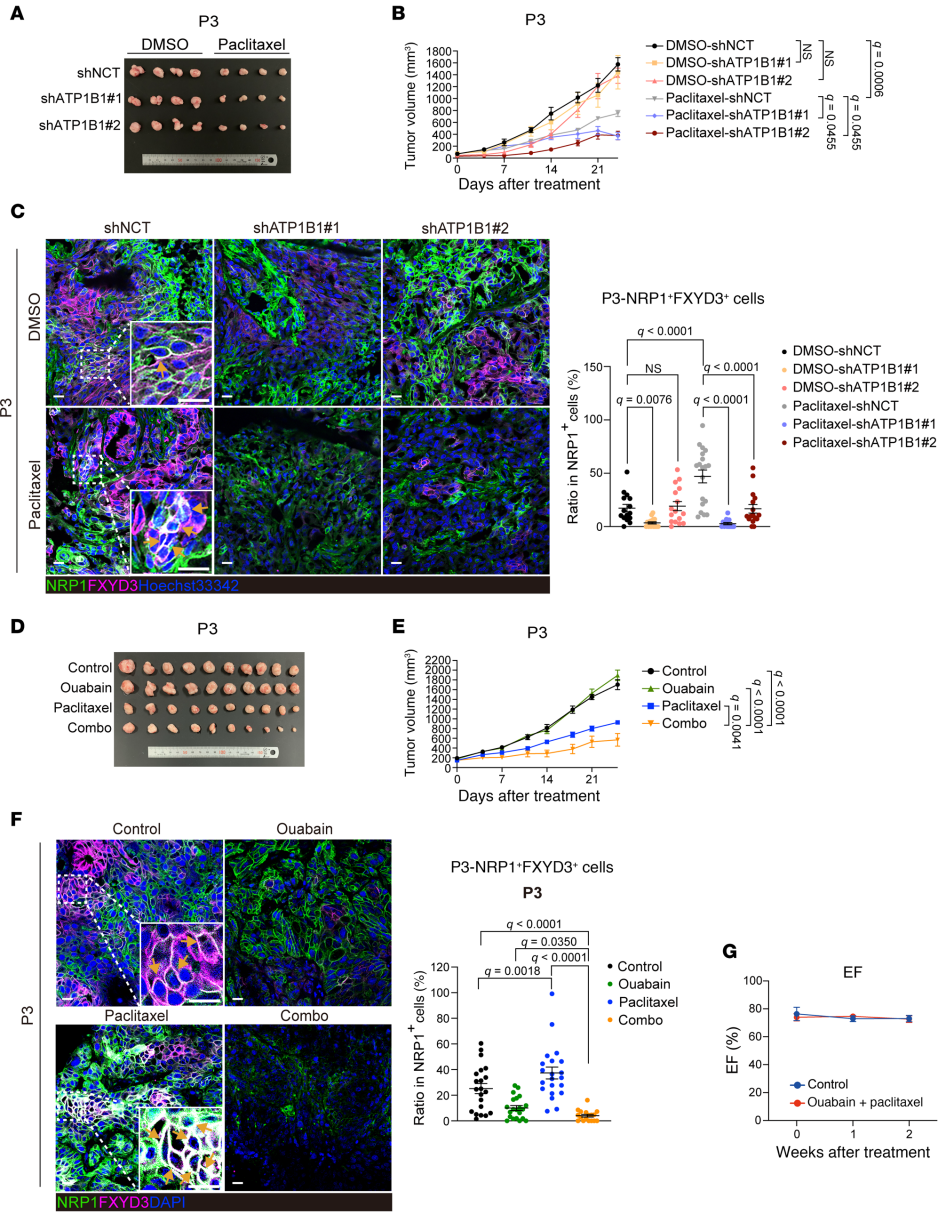
Knockdown of ATP1B1 sensitizes TNBC PDX tumors to paclitaxel treatment and decreases proportion of FXYD3-positive ancestor-like CSCs. (**A**) Images of tumors generated in mice. (**B**) Tumor growth curves during paclitaxel treatment. *n* = 4 for each condition of P3 PDX. Statistical significance was determined by 2-way ANOVA with 2-stage linear step-up procedure of Benjamini, Krieger, and Yekutieli post hoc tests. Results are shown as means ± SEM. (**C**) Left: Immunofluorescence staining of frozen tissues of PDX tumors using antibodies against NRP1 and FXYD3; nuclei were stained using Hoechst 33342. Yellow arrows indicate cells double-positive for NRP1 and FXYD3. Scale bars: 20 μm. Right: Quantification of the ratio (percent) of NRP1 and FXYD3 double-positive cells to total NRP1-positive cells. *n* = 16–20 random fields were collected for each condition. Outliers were excluded with the ROUT method before statistical analysis. Statistical significance was determined by 2-way ANOVA with 2-stage linear step-up procedure of Benjamini, Krieger, and Yekutieli post hoc tests. Results are shown as means ± SEM. (**D**) Images of tumors generated in mice. Combo, combination of paclitaxel and ouabain. (**E**) Tumor growth curves during paclitaxel treatment. *n* = 8 for each condition of P3 PDX. Statistical significance was determined by 2-way ANOVA with 2-stage linear step-up procedure of Benjamini, Krieger, and Yekutieli post hoc tests. Results are shown as means ± SEM. (**F**) Left: Immunofluorescence staining of paraffin tissues of PDX tumors using antibodies against NRP1 and FXYD3; nuclei were stained using DAPI. Yellow arrows indicate cells double-positive for NRP1 and FXYD3. Scale bars: 20 μm. Right: Quantification of the ratio (percent) of NRP1 and FXYD3 double-positive cells to total NRP1-positive cells. *n* = 21 random fields were collected for each condition. Outliers were excluded with the ROUT method before statistical analysis. Statistical significance was determined by 2-way ANOVA with 2-stage linear step-up procedure of Benjamini, Krieger, and Yekutieli post hoc tests. Results are shown as means ± SEM. (**G**) Quantification of the ejection fraction (EF) by transthoracic echocardiography. *n* = 7 mice for each condition. Statistical significance was determined by unpaired, 2-tailed Student’s *t* test. Results are shown as means ± SEM.

Next, we inoculated immunodeficient mice with *ATP1B1*-knockdown cancer cells and treated them with paclitaxel. *ATP1B1* knockdown did not affect tumorigenesis in the PDX derived from P3 sample, whereas treatment with paclitaxel inhibited tumorigenesis without significant body weight loss (Figure 7, A and B, and Supplemental Figure 9A). The combined treatment with paclitaxel and *ATP1B1* knockdown markedly inhibited tumorigenesis and led to tumor regression. *ATP1B1* knockdown or paclitaxel treatment significantly decreased tumorigenesis in the PDX derived from P5 sample without body weight loss (Supplemental Figure 9, B–D). Furthermore, combined treatment with paclitaxel and *ATP1B1* knockdown completely suppressed tumorigenesis.

Next, we evaluated antibodies against NRP1, IGF1R, and FXYD3 using immunohistochemistry based on the expression data of each protein using FACS (Supplemental Figure 10A). Cells positive for NRP1 (NRP1^med^ and NRP1^hi^ cells) or IGF1R (IGF1R^med^ and IGF1R^hi^ cells) and cells positive for FXYD3 (FXYD3^med^ and FXYD3^hi^ cells) were more abundant among P3 patient-derived cancer cells than in P6 and P1 patient-derived cells, respectively (Supplemental Figure 10A). Consistently, immunohistochemistry using anti-NRP1 and anti-IGF1R antibodies stained more cells in P3 than in P6 samples, whereas anti-FXYD3 antibodies stained more cells in P3 than in P1 samples. These antibodies can, thus, be appropriately used for immunohistochemistry. We observed that cells double-positive for NRP1 and FXYD3 (ancestor-like CSC enriched) among the NRP1-positive cells or cells double-positive for IGF1R and FXYD3 (ancestor-like CSC enriched) among the IGF1R-positive cells were increased in cancer tissues after paclitaxel treatment, whereas they were diminished in *ATP1B1*-knockdown cancer tissues (Figure 7C and Supplemental Figure 10B). Furthermore, cells positive for GCLC were significantly more frequent among NRP1 and FXYD3 (ancestor-like CSC enriched) or IGF1R and FXYD3 (ancestor-like CSC enriched) double-positive cells than among other cell populations in the PDX tumors (Supplemental Figure 10, C and D). Paclitaxel treatment increased the number of cells triple-positive for NRP1, FXYD3, and GCLC or for IGF1R, FXYD3, and GCLC, whereas *ATP1B1* knockdown suppressed their enrichment (Supplemental Figure 10, E and F). Collectively, we demonstrated that the inhibition of *ATP1B1* expression decreases the redox state and alleviates drug resistance in ancestor-like CSCs in vitro and in vivo.

Finally, we tested whether ouabain could enhance the drug response in vivo. Immunodeficient mice were inoculated with P3 cells and then treated with or without paclitaxel or ouabain. Notably, combined treatment with ouabain markedly increased sensitivity to paclitaxel without body weight loss (Figure 7, D and E, and Supplemental Figure 11A). We observed that the cells double-positive for NRP1 and FXYD3 (ancestor-like CSC enriched) were increased by paclitaxel treatment and they were significantly diminished by combined treatment with ouabain in cancer tissues (Figure 7F). Moreover, we escalated the doses of ouabain up to 3-fold (4.5 mg/kg), and tumorigenesis was inhibited in a dose-dependent manner without any observed body weight loss (Supplemental Figure 11, B–D). To assess potential cardiotoxicity while administering a combination treatment of ouabain and paclitaxel, we evaluated the cardiac functions of mice through echocardiography. Our observations revealed no significant alterations in left ventricle ejection fraction or left ventricle dilation, suggesting the absence of cardiotoxicity (Figure 7G and Supplemental Figure 11, E and F). Hence, we have provided proof of concept that inhibiting the Na^+^/K^+^ pump can ameliorate drug resistance in vivo without inducing cardiotoxicity.

### Ancestor-like CSCs are enriched after NAC and correlate with poor clinical prognosis.

**Figure 8:**
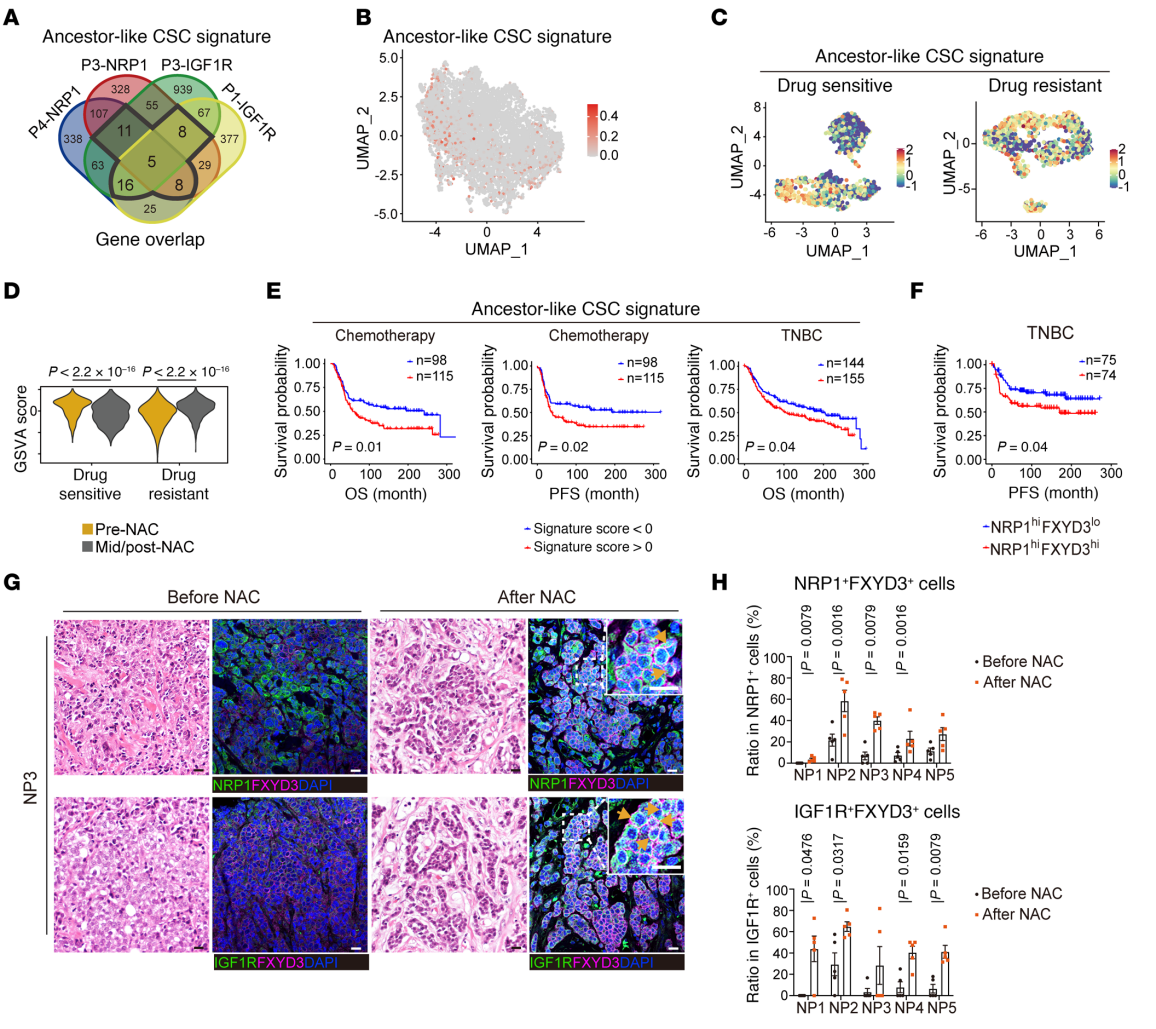
Ancestor-like CSCs are related to poor clinical prognosis. (**A**) Forty-eight genes (outlined by thick black lines) from the Venn diagram shown in Figure 3A were upregulated in quiescent clusters across 3 of 4 samples and selected as the ancestor-like CSC signature genes. (**B**) UMAP visualization of scRNA-Seq data from PDX models shown in Figure 1B colored using GSVA score of the ancestor-like CSC signature. (**C** and **D**) UMAP visualization of single-nucleus RNA-Seq data from NAC-sensitive or NAC-resistant TNBC cancer tissues shown in Figure 1D colored using GSVA score for the ancestor-like CSC signature and compared between pre- and mid-/post-treatment subgroups (**C**). Wilcoxon’s rank sum test (**D**) was used to determine significant *P* value. (**E**) Kaplan-Meier survival analysis between high (>0) and low (<0) subgroups of the ancestor-like CSC signature score in METABRIC cohort of breast cancer patients who received chemotherapy (not hormone therapy; *n* = 213) or belonged to TNBC subtype (*n* = 299). OS, overall survival; PFS, progression-free survival. (**F**) Kaplan-Meier survival analysis between NRP1^hi^FXYD3^lo^ and NRP1^hi^FXYD3^hi^ groups in METABRIC cohort of TNBC breast cancer patients. *n* = 149. Medians were used for cutoff value. *P* value was obtained using log-rank test. (**G**) Representative images of H&E staining and immunofluorescence staining of paired tumor samples of pre- and post-NAC from patients with TNBC, using antibodies against FXYD3 and NRP1 or IGF1R. Nuclei were stained using DAPI. Yellow arrows indicate cells double-positive for NRP1 and FXYD3 (top) and cells double-positive for IGF1R and FXYD3 (bottom). Scale bars: 20 μm. (**H**) Quantification of the ratio (percent) of the cells double-positive for NRP1 and FXYD3 to total NRP1-positive cells (top) and the cells double-positive for IGF1R and FXYD3 to total IGF1R-positive cells (bottom). *n* = 5 random fields were collected for each condition. Statistical significance was determined by 2-tailed Mann-Whitney *U* tests. Results are shown as means ± SEM.

We selected 48 commonly upregulated genes across 3 or 4 samples derived from quiescent cell clusters of the individual samples and designated these as our ancestor-like CSC signature genes (Figure 8A and Supplemental Table 5). Analysis of scRNA-Seq data obtained from our PDX models shows cells with ancestor-like CSC signature genes (Figure 8B). The expression of these signature genes after NAC was significantly upregulated in chemotherapy-resistant cancer tissues, whereas it was reduced in normal epithelial cells derived from the chemotherapy-sensitive cancer tissues (37) (Figure 1D and Figure 8, C and D). Analysis of bulk RNA-Seq data from the Molecular Taxonomy of Breast Cancer International Consortium (METABRIC) cohort showed that patients undergoing chemotherapy (not hormone therapy) or those with TNBC subtype with a greater population of ancestor-like CSCs have poor prognosis (54) (Figure 8E). Furthermore, TNBC patients with NRP1^hi^- and FXYD3^hi^-expressing tumors showed poorer prognosis than those with NRP1^hi^ but FXYD3^lo^ tumors (Figure 8F).

**Figure 9:**
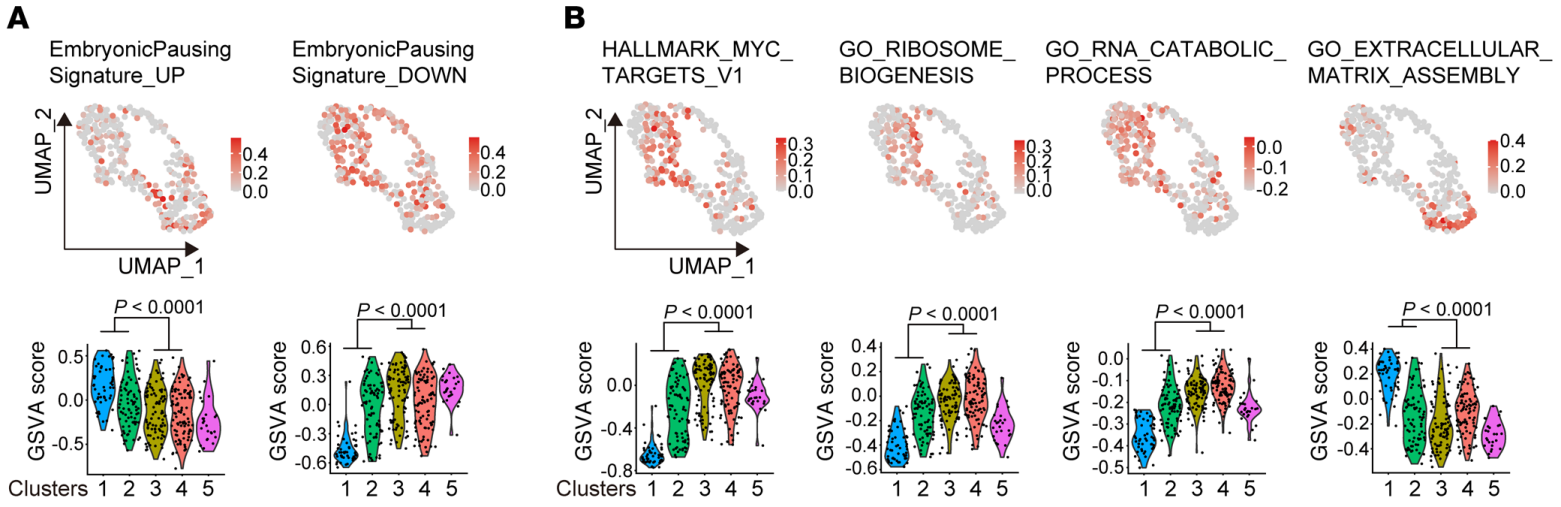
Ancestor-like CSCs possess features of DTPs. (**A** and **B**) Top: GSVA score of reported enriched gene sets in DTPs projected onto the UMAP derived from the SMART-seq data shown in Figure 2C. Bottom: Violin plots of GSVA score for each cluster. Statistical significance was determined by moderated *t* tests, and *P* value was adjusted by false discovery rate.

Next, we conducted immunohistochemistry assays to analyze the ratio of NRP1 and FXYD3 double-positive cells (ancestor-like CSC enriched) among the NRP1-positive population or the ratio of IGF1R and FXYD3 double-positive cells (ancestor-like CSC enriched) among the IGF1R-positive population in pre- and post-NAC cancer tissues derived from 5 TNBC patients with partial response to NAC (Supplemental Table 3). The ancestor-like CSC–enriched population was greatly increased after NAC treatment (Figure 8, G and H). Therefore, ancestor-like CSCs are resistant to chemotherapy and may lead to relapse. These findings raise the possibility that ancestor-like CSCs phenotypically overlap with DTPs. It has been recently reported that breast cancer DTPs emerge as a result of an embryonic diapause-like adaptation to the stress induced by chemotherapy (14). Myc downregulation leads to downregulation of ribosome and RNA metabolism in DTPs with a concurrent upregulation of extracellular matrix assembly. Interestingly, we found that the upregulated genes in the embryonic diapause were also upregulated in cluster 1 and 2 cells, which comprised the ancestor-like CSCs; similarly, the downregulated genes in embryonic diapause were downregulated in the ancestor-like CSCs (55) (Figure 9A). Furthermore, we found that Myc target gene expression and metabolism of ribosomes and RNAs were downregulated in ancestor-like CSCs compared with those in alveolar progenitor–like CSCs, whereas extracellular matrix assembly was upregulated in these cells (Figure 9B). Together, these observations suggest that ancestor-like CSCs phenotypically overlap with the DTPs derived from TNBC.

We used UMAP on the scRNA-Seq data of 11,295 individual cells, including all the cells derived from our PDX models. This analysis showed that these cells could be grouped into several clusters (Supplemental Figure 12A, left). *KRT19* (tumor epithelial cells), *PTPRC* (CD45; immune/hematopoietic cells), and *PDGFRB* (stromal cells) were highly expressed by cells in different clusters (Supplemental Figure 12B). The *KRT19*-positive tumor cell cluster comprised cells derived from all 3 samples (Supplemental Figure 12, A [middle] and B). Moreover, some cells expressing ancestor-like CSC signature genes were also present in this *KRT19*-positive cluster, whereas very few of these cells were present in the other clusters (Supplemental Figure 12A, right); this indicates that ancestor-like CSC signature genes are specifically expressed in a few tumor cells but not in other cells in the tumor microenvironment. We next examined the enriched pathways in tumor cells expressing high and low levels of ancestor-like CSC signature genes using GO analysis (Supplemental Figure 12C). Cells expressing ancestor-like CSC signature genes at high levels showed upregulation of stem cell maintenance, oxidant detoxification, negative regulation of Ca^2+^ channel, and sodium ion homeostasis pathways, which are all reasonable signaling pathways to be activated in ancestor-like CSCs. In turn, cells expressing ancestor-like CSCs at low levels showed upregulation of cell fate commitment, cell maturation, and cell fate specification pathways, which are common in differentiated cells. Next, we used UMAP to analyze publicly available scRNA-Seq data of 1,107 individual cells derived from 6 TNBC samples (56) (Supplemental Figure 12D, left). The *KRT19*-positive tumor cell cluster comprised cells derived from all samples (Supplemental Figure 12, D [middle] and E). Notably, some ancestor-like CSC signature–expressing cells were in the tumor cell cluster, but were scarce in the other clusters (Supplemental Figure 12, D [right] and E). The GO analysis revealed that the pathways upregulated in cells expressing ancestor-like CSC signature genes at high or low levels were similar to those in our PDX models (Supplemental Figure 12, C and F). Together, these results corroborate that the ancestor-like CSC signature genes specifically represent the traits of these cells in the tumor microenvironment.

## Discussion

In this study, we successfully identified ancestor-like CSCs that expressed high levels of FXYD3 in the extremely heterogeneous CSC population in TNBC. Ancestor-like CSCs are a major cause of therapeutic resistance and possess common vulnerability mechanisms shared across TNBC tissues. The identification of ancestor-like CSCs using FACS, along with single-cell gene expression profiling, facilitated their detailed characterization.

The ancestor-like CSCs possess mammary stem– or luminal progenitor–like traits and are quiescent, whereas CSCs with low expression of FXYD3 possess traits of alveolar progenitors that develop specifically during pregnancy under physiological conditions (19). The intrinsic proliferation ability of these cells enables the rapid growth of the tumor mass, reminiscent of the rapidly developing pregnant mammary tissues with densely crowded acinar structures. Along with cell lineage development, ancestor-like CSCs give rise to alveolar progenitor–like CSCs and non-CSCs. Few cells from each cell population dedifferentiate in the reverse direction. Therefore, cancer cells exhibit cellular plasticity and reversible differentiation and dedifferentiation state. Hence, chemotherapy-treated FXYD3^lo^ CSCs may lead to the generation of more FXYD3^hi^ cells than those that are untreated. Further studies are warranted to explore this possibility.

ROS-induced glutathionylation of ATP1B1 cysteine residues inhibits Na^+^/K^+^ pump activity (23). However, FXYD3 can prevent this event by undergoing glutathionylation instead of ATP1B1, thereby maintaining the activity of the Na^+^/K^+^ pump. Herein, we show that both *FXYD3a* and *FXYD3b* isoforms are expressed in ancestor-like CSCs. Since the cysteine residues that can undergo ROS-induced glutathionylation exist in the cytoplasmic domains of both isoforms (53), it is possible that both FXYD3 isoforms can protect the ATP1B1 subunit from glutathionylation. FXYD3 maintains the activity of the Na^+^/K^+^ pump to establish a decreased intracellular Ca^2+^ concentration and balanced redox status; this confers resistance to stressful conditions such as chemotherapy, since a pivotal consequence of chemotherapy, paclitaxel included, is ROS elevation, leading to cell death in numerous cancer types (57). Cardiac glycosides inhibit the Na^+^/K^+^ pump in cardiac myocytes and lead to an increase in intracellular concentration of Na^+^, which is then pumped out of the cytoplasm through the cooperative Na^+^/Ca^2+^ exchanger (NCX) on the plasma membrane to adjust the ion balance between the extracellular and intracellular spaces (49). The resultant increase in intracellular Ca^2+^ concentration induces contraction of muscle fibers, resulting in the improvement of cardiac function. However, in other cells, ROS are easily generated and may mitigate drug resistance at high Ca^2+^ concentrations. Ouabain and digoxin, at submicromolar concentrations, were reported to bind to Na^+^/K^+^-ATPase and stimulate NADPH oxidase (a complex of superoxide-generating enzymes) in vitro and increase ROS levels (58). Since we found that ouabain and digoxin can inhibit the proliferation of patient-derived cancer cells at submicromolar concentrations in vitro, it is thus possible that these mechanisms contributed to the inhibition of cancer cell proliferation in our experiments as well. Cardiac glycosides also act as senolytic drugs (28, 29), as increased ROS production due to increased Ca^2+^ concentration damages senescent cells. Our findings provide evidence that ancestor-like CSCs and senescent cells share common maintenance mechanisms dependent on the activity of the Na^+/^K^+^ pump.

The presence and properties of DTPs in a variety of cancer types have been widely investigated (13–17, 59–61). In DTPs or pre-DTPs, primed cells poised to become DTPs undergo a diapause-like cell state in response to drug treatment. Changes in cell states between regular cancer cells and DTPs are reversible and share properties with senescent cells. We observed that ancestor-like CSCs share numerous properties with DTPs. It appears that DTPs possess different traits depending on the cancer type or subtype from which they are derived. In TNBC, embryonic diapause-like adaptation occurs in the DTPs (14). Downregulation of the Myc pathway is crucial, and is associated with the downregulation of ribosome and RNA metabolism and upregulation of extracellular matrix assembly. Notably, a similar cellular state is observed in the ancestor-like CSCs defined in the current study. Therefore, DTPs and ancestor-like CSCs substantially overlap in TNBC cell populations.

In normal tissues, a small population of tissue-specific stem cells gives rise to progressive cell differentiation, with a highly ordered hierarchy (62). However, compelling evidence indicates the existence of a heterogeneous CSC population in cancer tissues, which includes ancestor-like CSCs with cell-of-origin traits, and one-step-differentiated, highly proliferative alveolar progenitor–like CSCs with a variety of functional properties as reported in many studies, including ours (5, 6, 8–11, 63, 64). These heterogeneous CSCs survive, differentiate, and grow with flexible plasticity, but remain substantial, allowing them to survive under harsh conditions.

FXYD3 is a multifunctional protein. In normal cells, FXYD3b is expressed more in differentiated cells, whereas FXYD3a is expressed more in undifferentiated cells (23). FXYD3a reduces the Na^+^ affinity of the Na^+^/K^+^ ATPase, compared with FXYD3b. This difference in effect may lead to variations in intracellular Na^+^ concentration during normal differentiation processes (23, 65). Both FXYD3 isoforms were shown to induce a hyperpolarization-activated chloride current in *Xenopus* oocytes (66). Another study reported that TGF-β signaling negatively regulates FXYD3 expression through the transcription factor ZEB/dEF1 (67). In hormone receptor–positive breast cancer, FXYD3 interacts with Src tyrosine kinase to recruit phosphatidylinositol-3-kinase, thereby stimulating the proliferation of CSCs (68). Furthermore, FXYD3 is expressed in some populations of NRP1^lo^ and IGF1R^lo^ non-CSCs. Herein, siRNA-mediated inhibition of both *FXYD3a* and *FXYD3b* in patient-derived breast cancer cells resulted in a significant decrease in bulk cell proliferation. FXYD3 expression is upregulated in bulk cancer cells such as those in breast, pancreas, colon, prostate, and endometrium (52, 69–71). However, how FXYD3 contributes to the proliferation of differentiated non-CSCs and which isoform plays a greater role in patient-derived breast cancer cell proliferation remain largely unknown. As a potential therapeutic target, it is favorable that targeting FXYD3 can inhibit both pro-tumorigenic functions: maintenance of Na^+^/K^+^ pump activity and stimulation of cell proliferation.

Recent reports suggest that ATP1B1 can associate with myotonic dystrophy kinase–related Cdc42-binding kinase α (MRCAα), activating signaling pathways that regulate tight junction assembly (72). Thus, FXYD3 and ATP1B1 may cooperatively maintain the activity of the Na^+^/K^+^ pump and, consequently, support drug resistance in ancestor-like CSCs while working independently in other cancer cells for other functions. This complex interplay with multiple proteins appears to contribute to cancer cell proliferation and tumorigenesis. Further experiments are necessary to unravel the molecular mechanisms underlying this phenomenon, as FXYD3 remains a promising target for cancer therapy to improve treatment outcomes.

There are several strategies to target the Na^+^/K^+^ pump. Clinical trials of combined therapies with cardiac glycosides are warranted, since they are commonly used drugs (27). Recently, a deeper understanding of genomic and molecular characteristics of TNBC has enabled its classification into the following subtypes with personalized treatment options: *BRCA1/2*-mutant tumors; tumors with BRCAness, sharing molecular features with *BRCA1/2*-mutant tumors; and PD-L1–high tumors (73). However, there remains the risk of residual disease after NAC. Treatment with trastuzumab deruxtecan, an antibody-drug conjugate consisting of a humanized anti-HER2 antibody linked to a topoisomerase I inhibitor, results in significantly better overall survival of patients with HER2-low (but not HER2-absent) metastatic breast cancer (74, 75). However, a significant population of patients with TNBC are HER2 negative (76). An antibody-drug conjugate against FXYD3 or ATP1B1 would likely target both NAC-resistant CSCs/DTPs and non-CSCs regardless of the HER2 status to contribute to effective TNBC therapy.

Altogether, we provide compelling evidence that the ancestor-like CSC and DTP cell populations overlap, and proof of principle to target the Na^+^/K^+^ pump to treat TNBC.

## Methods

### Statistics.

Statistical analyses were performed in R environment (version 4.1.2; https://www.r-project.org/) or GraphPad Prism software (version 9.3.1). Statistical methods and details are described in the figure legends and in Supplemental Data 1. Briefly, for normally distributed data, the significance was calculated with the unpaired, 2-tailed Student’s *t* test. Comparisons between more than 2 groups was performed using 1-way ANOVA or 2-way ANOVA with Bonferroni’s post hoc analysis. For non-normally distributed data, the significance was calculated with the Mann-Whitney *U* test (2-group comparisons) or Dunn’s multiple-comparison tests between groups (>2 groups). The in vivo tumorigenesis data were analyzed by 2-way ANOVA with 2-stage linear step-up procedure of Benjamini, Krieger, and Yekutieli post hoc tests.

Signature scores were calculated as the average of normalized expression (*z* score method) of all genes in the list or as gene set variation analysis (GSVA) scores (see Supplemental Methods). Signature score greater than 0 and less than 0 indicates high and low scores, respectively. NRP1^hi^FXYD3^hi^, NRP1^hi^FXYD3^lo^, FXYD3^hi^, and FXYD3^lo^ subgroups were defined according to the medians of *NRP1* and *FXYD3* gene expression. Kaplan-Meier curves were generated using the ggsurvplot function with the survival (version 3.3.1) (77) package in R. Log-rank test was used to determine significant *P* values.

The *x* and *y* axes for the UMAP figures are described in Supplemental Data 2.

Tumor spheroid-forming ability was analyzed by extreme limiting dilution assay (ELDA) software (https://bioinf.wehi.edu.au/software/elda/) (78). Data are presented as the mean ± SD or the mean ± SEM.

### Study approval.

Fresh breast cancer tissues were obtained from patients undergoing surgical resection or biopsy at Kanazawa University Hospital or University of Tokyo Hospital. Sample use was approved by the IRBs of the Cancer Research Institute of Kanazawa University, Kanazawa University Hospital, the Institute of Medical Science of The University of Tokyo, Minami-machida Hospital, and University of Tokyo Hospital (approval no. 331-12). Written informed consent was obtained from all patients prior to inclusion in this study. All experiments were conducted according to the principles established by the Declaration of Helsinki.

Paraffin tissue sections from 5 patients with breast cancer who underwent both biopsy and mastectomy between 2014 and 2018 were obtained from Kanagawa Cancer Center. Frozen sample correction was conducted according to the guidelines of the Declaration of Helsinki, with the approval of the Institutional Review Board of Kanagawa Cancer Center (approval no. 28KEN4) and Kanazawa University (approval no. 331-12).

Female NOD.CG-*PRKDC^SCID^IL2RG^TM1WJL^*/SZJ (NSG) mice (Charles River Laboratories) were handled according to the guidelines of the Institute for Experimental Animals, Kanazawa University. Animal experiments were approved by the committee for animal research of Kanazawa University (approval no. AP-194036).

### Publicly available data set.

In this study, publicly available data were analyzed. *FXYD3* levels were compared between invasive breast carcinoma and healthy tissues on the basis of The Cancer Genome Atlas (TCGA) data sets obtained from the University of Alabama at Birmingham Cancer Data Analysis Portal (UALCAN) (http://ualcan.path.uab.edu/index.html) (79). The transcriptome profiles of breast tumor tissues were obtained from the Gene Expression Omnibus (GEO) database data sets GSE1456 (80) and GSE7378 (81) and the METABRIC cohort (54), as well as from patients with TNBC who participated in the PROMIX trial (GEO GSE87455; ref. 38).

### Data availability.

All data generated or analyzed during the present study, including source data, can be found in the article or in the Supplemental Supporting Data Values file. The accession numbers for the 10X Genomics–based scRNA-Seq data and for the Fluidigm C1–based scRNA-Seq data reported in this study are DNA Data Bank of Japan (DDBJ) Sequence Read Archive DRA014426 and NBDC Human Database JGAS000305/JGAD000416, respectively. The scripts and pipelines used for data preprocessing are described in Supplemental Methods; no new code or algorithms were generated.

Additional patient data are available upon request with a transfer agreement or collaborative arrangement.

## Author contributions

ML performed most of the experiments, analyzed data, performed bioinformatics analysis, and contributed to the writing of the manuscript. TN and YT performed many experiments and analyzed data. TY, S Sato, and YM performed pathological analysis of patient samples. MN and SO performed exome sequencing of the PDX samples. HH, TS, AK, YS, and KO performed single-cell transcriptome analysis using the Fluidigm C1 system. TH performed several experiments. YW, TH, and DS analyzed data. AS, MY, SI, MI, TO, MT, and KT provided the clinical samples. TA, LAEN, and RN performed bioinformatics analysis. DCV contributed to discussions and writing of the manuscript. XC, CCL, and S Sugano contributed to expert discussions. YU, TC, and HA performed single-cell transcriptome analysis using the 10X Genomics platform. MK and SN performed echocardiographic studies. AT supervised the study. KW and TF analyzed single-cell transcriptomes of the pre- and mid- to post-NAC patient samples. NG conceptualized the study, contributed to the study design, and drafted and finalized the manuscript.

## Supplementary Material

Supplemental data

Supplemental data set 1

Supplemental data set 2

Supplemental table 5

Supporting data values

## Version 1 11/15/2023

Electronic publication
