# Supplementary material for: FXYD3 functionally demarcates an ancestral breast cancer stem cell subpopulation with features of drug-tolerant persisters
Source: J Clin Invest. 2023 Nov 15;133(22):e166666. doi: 10.1172/JCI166666 (PMC10645391; doi:10.1172/JCI166666)
Supplement: Supplemental data [file jci-133-166666-s190.pdf]

Supplemental Information

FXYD3 functionally demarcates an ancestral breast cancer stem cell subpopulation with features of drug tolerant persisters

Mengjiao Li, Tatsunori Nishimura, Yasuto Takeuchi, Tsunaki Hongu, Yuming Wang, Daisuke Shiokawa, Kang Wang, Haruka Hirose, Asako Sasahara, Masao Yano, Satoko Ishikawa, Masafumi Inokuchi, Tetsuo Ota, Masahiko Tanabe, Kei-ichiro Tada, Tetsu Akiyama, Xi Cheng, Chia-Chi Liu, Toshinari Yamashita, Sumio Sugano, Akinori Kanai, Yutaro Uchida, Tomoki Chiba, Hiroshi Asahara, Masahiro Nakagawa, Shinya Sato, Yohei Miyagi, Teppei Shimamura, Luis Augusto Eijy Nagai, Akinori Kanai, Manami Katoh, Seitaro Nomura, Ryuichiro Nakato, Yutaka Suzuki, Arinobu Tojo, Dominic C. Voon, Seishi Ogawa, Koji Okamoto, Theodoros Foukakis, Noriko Gotoh

Supplemental Materials and Methods

Supplemental Figures 1-12

Supplemental Tables 1-5

Supporting Data 1 and 2

Supplemental Materials and Methods

Cell Lines

Human breast cancer cell lines HCC38 and MDA-MB-231 were obtained from American Type Culture Collection (ATCC). HEK293T cells were a kind gift from Dr. Toshio Kitamura (The University of Tokyo). HCC38 was maintained in Roswell Park Memorial Institute (RPMI) 1640 Medium (Nacalai Tesque, 30264-56). MDA-MB-231 and HEK293T cells were maintained in Dulbecco's modified Eagle's medium (DMEM) (Nacalai Tesque, 08458-45), supplemented with 10% fetal bovine serum (Thermo Fisher Scientific, 26140-079), 100 U/mL penicillin, and 100 µg/mL streptomycin (Nacalai Tesque, 26253-84) in a humidified atmosphere at 37 °C in 5% CO_2 in adherent condition.

Patient-derived Xenografts (PDXs)

PDX models were established as previously described (1). Briefly, breast cancer tissues obtained from patients were cut into 1-mm squares, and five pieces were suspended in Matrigel (Corning, 354234) to produce 50 µL of the cell mixture. Five pieces per site, or cells expressing the indicated constructs, were subsequently injected into the mammary fat pads of NSG mice. When tumors reached >1,000 mm^3, the mice were killed. Tumor tissues (< 10 passages) were used for analysis.

10× Genomics-based Single-cell RNA Sequencing (scRNA-seq)

Tumor tissues derived from the PDXs (P1, P2, and P3) were subcutaneously transplanted in 6–7-week-old female NOD.Cg-$PRKDC^{SCID}IL2RG^{TM1SUG}$/ShiJic (NOG) mice (Central Institute for Experimental Animals, Kawasaki, Japan) with Matrigel (Corning, 354234). Breast cancer tissues from PDXs were collected 8 (P1, P2) or 5 (P3) weeks after transplantation. Fresh breast cancer tissues were digested into single cells by collagenase (FUJIFILM Wako, 032-22364), and red blood cells were removed using RBC lysis buffer (Thermo Fisher Scientific, 00-4333-57). Single, live cells were loaded into 10× Chromium Single Cell Capture Chip, followed by single-cell capture, reverse transcription, and library preparation, according to the manufacturer's protocol. The prepared library was sequenced on an Illumina HiSeq (P1, P2) or NovaSeq (P3) platform, according to manufacturers' protocols. For analyzing all types of cells in tumor tissues (Supplemental Figure 12), the output fastq files were processed using the *cellranger count* function of Cell Ranger (version: 2020-A, July 7, 2020; https://support.10xgenomics.com/single-cell-gene-expression/software/overview/welcome). The human transcriptome reference package used was obtained from 10× Genomic website.

Discrimination of Human Tumor Cells

To distinguish the data originated from grafted (human tumor) cells from those of host (mouse) cells, XenoCell package was exploited (2). First, using the *generate_index* function, mouse and human genome indexes were generated based on the mm10 and hg38 reference genomes, respectively. Next, the reads were classified into either mouse or human transcripts using the *classify_reads* function, and graft-specific cellular barcodes were extracted using the *extract_cellular_barcodes* function, with cellular barcodes, which contained 70–100% of graft reads, being extracted. The output fastq files were processed using the *cellranger count* function of Cell Ranger. The human transcriptome reference package used was obtained from 10× Genomic website. Finally, a unique molecular identified (UMI) count matrix was generated for downstream analysis.

The following processes were performed using Seurat vignettes of R package (version 4.1.0)(3).

Quality Control

Genes expressed in less than five cells were eliminated. Cells with < 500 or > 5,000 genes, or with a fraction of mitochondrial genes > 20% were discarded to eliminate low-quality cells with mitochondrial contamination.

Normalization

Gene transcript counts were normalized using *NormalizeData* within the LogNormalize method, in which gene transcript counts for each cell are divided by the total counts for that cell, multiplied by a scale factor (scale.factor = 10,000 by default), and then natural-log transformed using log1p method. A total of 2,000 highly variable genes were selected for further analysis.

Clustering

To reduce data dimension, principal component analysis (PCA) was applied using default parameters. Best dimensionality was determined using *JackStrawPlot* and *ElbowPlot* functions. Dim = 20 was used for integrated data and dim = 10 for P1, P2, and P3. Graph-based clustering approach was used to cluster cells using *FindNeighbours* and *FindClusters* functions. With respect to the parameter used for the *FindClusters* function, res = 0.15 (Figure 1B), res = 0.1 (Supplemental Figure 12A) were used for integrated data and res = 0.5 for P1, P2, and P3 data. *RunUMAP* and *DimPlot* functions were used to perform nonlinear dimension reduction and to visualize the data, respectively.

Data Integration

Individual data of 10× scRNA-seq PDX (P1, P2, and P3) samples were integrated using *FindIntegrationAnchors* and *IntegrateData* functions (3) introduced in Seurat with default parameters.

Heatmap of Top10 Genes for Each Cluster

Upregulated genes (marker genes) of each cluster were identified using the FindAllMarkers function (min.pct = 0.25, logfc.threshold = 0.2, test = wilcox for Wilcoxon rank sum test), and the top10 upregulated genes of each cluster were shown in heatmap.

Gene Set Variation Analysis (GSVA)

The mammary stem cell signature contains all the genes which are specifically upregulated in cluster 15 compared with those in all other clusters (clusters 1-14), described in publicly available single-cell data of mouse mammary glands (4) (Supplemental Table 5). Similarly, the luminal and alveolar progenitor signatures contain all the genes which are specifically upregulated in cluster 6 and 10, respectively, compared with those in all the other clusters (4)(Supplemental Table 5). GSVA algorithm (GSVA R package [version 1.42.0]), a Kolmogorov-Smirnov-like rank statistic based on kernel estimation of the cumulative density (5), was employed to calculate signature enrichment scores for each single cell. To define differentially enriched gene sets, limma difference analysis (*limma* v3.50.1, R package)(6) was performed between ancestor-like CSC signature gsva score > 0 and < 0 groups of integrated data of human tumor cells (Supplemental Figure 12A) (moderate *t*-tests, *P*-value was adjusted to the false discovery rate).

Data Analysis of Single-nucleus RNA seq (snRNA-seq) of Longitudinal TNBC Patient Samples with NAC

SnRNA-seq data were acquired from 20 tumor samples of eight TNBC patients included in the PROMIX trial (NCBI Sequence Read Archive accession no. SRP114962)(7). Clonal evolution analyses based on longitudinal samples (pre/mid/post NAC) indicated that clonal persistence (drug resistant) and extinction (drug sensitive) occurred in four patients each. Data matrix of log-transformed transcript per million (log[TPM/10+1]) value was used to conduct downstream analyses, filtered to include genes that were expressed in at least 30% of the cells. The data of 20 libraries were integrated using *Harmony* (8) from Seurat–, according to sample label. UMAP dimensionality reduction was conducted based on the top principal components, and breast epithelial cells were identified by clusters with mean gene expression (i.e. tumor cell: EPCAM; normal breast cell: ACTA2, KRT18/19) with cutoff > 0.8. Two-way ANOVA with interaction tests was used to evaluate the GSVA score of immature mammary epithelial gene signatures, based on different time points (pre-treatment *vs.* mid/post-treatment) and response to NAC (resistant *vs.* sensitive). GSVA scores were calculated using bulk microarray gene expression data from the PROMIX trial (GSE87455), and the optimal cutoff GSVA score was determined by the maximally selected rank statistics when we evaluated the correlation between the luminal progenitor signature and event-free

survival using the *surv_cutpoint* function in the *surminer* R package (Supplemental Figure 1D).

Patient-derived Cancer Cell Culture and Single Cell Sorting for scRNA-seq of Breast CSCs

Patient-derived breast cancer cells were isolated from human tumor specimens or NSG PDXs as previously described (1). Briefly, Fresh breast cancer tissues from PDXs were dissociated into single cells by collagenase (Sigma Aldrich, C9407), and red blood cells were excluded by RBC lysis buffer (Sigma Aldrich,11814389001). Mouse cells were eliminated by FACS sorting using APC-conjugated H-2Kd/H-2Dd antibody (Miltenyi Biotec, 130-107-895) to recognizes mouse major histocompatibility class I (MHCI). Isolated lineage-negative (Lin^-) breast cancer cells were cultured on collagen-coated dishes (IWAKI) in the organoid medium (Supplementary Table 3)(9) or in EpiCult-C Human Medium Kit medium (STEMCELL Technologies, 05630) that includes a supplement mix, freshly prepared 1 μM hydrocortisone (STEMCELL Technologies, #07904), 2 mM L-Glutamine (Nacalai Tesque,13004-02), 100 U/ml penicillin, and 100 μg/ml streptomycin (Nacalai Tesque, 26253-84) in a humidified atmosphere at 37 °C in 5% CO_2, and the culture medium was changed every 3-4 days. When cells reached 80–90% confluence, they were dissociated by Accumax (Innovative Cell Technologies, AM105) for 5–10 min at 37 °C. Single cells were collected and resuspended with FACS buffer (phosphate-buffered saline, PBS; Nacalai Tesque,14249-24), including 2 mM EDTA (Nacalai Tesque, 15130-95) and 0.5% bovine serum albumin (BSA; Nacalai Tesque, 01863-48) at a density of approximately 1×10^7 cells/mL. Subsequently, cells were incubated with 10 μL of PE-conjugated NRP1 antibody (R&D systems, FAB3870P) or 20 μL of PE-conjugated IGF1R antibody (BD Biosciences, 555999) at 4 °C for 30 min, and washed twice with FACS buffer. Twenty microliter of cell viability solution (BD Biosciences, 555816) was used to eliminate dead cells. Cells were analyzed and $NRP1^{high}$ or $IGF1R^{high}$ cells were collected by flow cytometry cell sorter (BD FACSAria III).

Fluidigm C1-based scRNA-seq

$NRP1^{high}$ or $IGF1R^{high}$ single-cell capture and whole transcriptome amplification were performed using the Fluidigm's C1 system, which is commonly used for analysis of numerous genes in individual cells, along with the SMART-Seq v4 Ultra Low RNA Kit (Takara Bio, 6350525), according to the manufacturer's instructions. cDNA quantification was performed with the Quant-IT PicoGreen dsDNA Assay Kit (Thermo Fisher Scientific, P11496), and the samples were diluted to be 0.1–0.3 ng/μL. Then, Nextera XT DNA sample preparation Kit (Illumina, FC-131-1096) and IDT for Illumina Nextera DNA UD Indexes (Illumina, 20027213,

20027214, 20027215, 20027216) were used for dual indexing and amplification, according to the manufacturer's protocols. Samples were sequenced using an Illumina HiSeq3000 sequencing system.

RNA-seq Data Processing

Fastq files were aligned and quantified using Kallisto (version 0.45.0)(10) in relation to the human reference transcriptome obtained from the GENCODE database (version 30)(11). Gene transcript counts for each sample were summarized in a matrix.

After mapping reads to the human reference genome (GRCh38) using STAR v2.6,0a (12), The expression quantification of *FXYD3* isoforms (*hFXYD3a* mRNA, NM_005971 and *hFXYD3b* mRNA, NM_021910) was performed using the reference guided mode of StringTie version 2.2.1 (13).

Quality Control

Empty captures, dead cells, and cell aggregates were filtered out with records of microscope observation of C1 integrated fluidic circuit plates during the single-cell capture step. Genes expressed in less than five cells were removed. Then, cells with < 7,000 (P1-IGF1R^{high} and P3-NRP1^{high}) or < 10,000 (P3-IGF1R^{high} and P4-NRP1^{high}) genes, or with a fraction of mitochondrial genes > 5% for P1-IGF1R^{high}, > 12.5% for P3-NRP1^{high}, > 20% for P3-IGF1R^{high}, or > 15% for P4-NRP1^{high}, were discarded to eliminate low-quality cells with mitochondrial contamination.

Normalization

Normalization of the data was performed as described for 10× Genomics-based scRNA-seq data.

Clustering

Data clustering was performed as described for 10× Genomics-based scRNA-seq data. To reduce the dimensions of the Fluidigm C1-based scRNA-seq data, dim = 10 was used for integrated data; dim = 10 for P1-IGF1R^{high}, P3-IGF1R^{high}, and P4-NRP1^{high}; and dim = 30 for P3-NRP1^{high}. With respect to the parameters used in the *FindClusters* function, res = 0.9 was used for integrated data, res = 1.0 for P1-IGF1R^{high} and P3-IGF1R^{high}, res = 1.2 for P3-NRP1^{high}, and res = 0.9 for P4-NRP1^{high}.

Data Integration

Data from P1-IGF1R^{high}, P3-NRP1^{high}, P3-IGF1R^{high}, and P4-NRP1^{high} samples were integrated using *FindIntegrationAnchors* and *IntegrateData* functions with k.filter = 50 and k.weight = 50, respectively.

Pseudotime Trajectory Analysis

R package Monocle3 (version 1.0.0)(14) was used for pseudotime trajectory analysis by implanting dimension and cluster information obtained from the analysis with Seurat

pipelines. Root cells were defined as those that had the highest GSVA score of mammary stem cell or luminal progenitor signatures, as described in Figure 2E. *Plot_genes_in_pseudotime* function was used to visualize the dynamics of the genes, commonly upregulated in quiescent clusters of each individual data, along the pseudotime.

RNA verocity analysis

To perform RNA velocity analysis, we calculated spliced/unspliced ratio using Velocyto version 0.17.17 (15) and applied UniTVelo version 0.2.4 (16) with default parameters. We imported the clusters obtained in Seurat using the R package SeuratDisk version 0.0.0.9020 (https://github.com/mojaveazure/seurat-disk).

GSVA

Signature enrichment scores of 4,090 biological process gene sets (gene set size 15–500) from gene ontology (GO) and 50 hallmark gene sets, described in the molecular signature database (MSigDB)(17), quiescent stem cell signature (18) human mammary gland stem/progenitor signature (Basal-Luminal Cell_Human [BL1+BL2] , Alveolar Progenitor_Human [AP1+AP2+AP3+AP4]) (19) and Embryonic Pausing Signature (20) (Supplementary Table 5), were calculated for each cell using the *GSVA* R package, as described above. To define differentially enriched gene sets, limma difference analysis was performed between clusters 1–2 and 3–4 of integrated data of Fluidigm C1-based scRNA-seq (moderate *t*-tests, *P*-value was adjusted to the false discovery rate).

Differential Gene Expression Analysis

Upregulated genes (marker genes) of each cluster were identified using the *FindMarkers* function (one cluster *vs*. the other clusters; logfc.threshold = 0.2, test = wilcox for Wilcoxon rank sum test).

Cell Cycle Analysis

The cell cycle phase-specific gene signatures defined by Macosko *et al.* (21) and Oki *et al.* (22) were used to calculate phase-specific scores for G_0, G1S, G2M, M, MG1, and S, by using a scoring method developed by Tirosh *et al.* (23) and is implemented in Seurat as “AddModuleScore” function (ctrl.size =100). Phase-specific scores were normalized by Z-score method and each cell was assigned to a cell-cycle stage based on its highest Z-score.

Cell Cycle Regression Analysis

The effects of cell cycle-related genes (difference between G2M and S phases) were removed using the *vars.to.regress* function in Seurat vignettes of cell cycle regression alternate workflow, which was adapted for analysis of stem cell development.

Data Analysis of Single-cell RNA seq (scRNA-seq) of TNBC Patient Samples

ScRNA-seq data were acquired from tumor samples of 6 TNBC patients (Gene Expression

Omnibus (GEO), accession code GSE 118389) (24). The following processes were performed using *Seurat vignettes* R package. For quality control, cells with < 500 or > 10,000 genes were discarded. Normalization and clustering were performed as described above. Data from PT039, PT058, PT081, PT084, PT089 and PT126 patients were integrated by using *FindIntegrationAnchors* and *IntegrateData* functions with k.weight = 50, respectively. Dim = 10 was used for individual data and integrated data. Resolution = 0.2 was used for integrated data. Difference analysis between ancestor-like CSC signature gsva score > 0 and < 0 groups of integrated data of tumor cells (Supplemental Figure 12D) was performed as described above.

Tumor Spheroid Formation

Cells were cultured in the sphere formation medium that include DMEM/F12 medium (Thermo Fisher Scientific, 11330-032) supplemented with 20 ng/mL epidermal growth factor (EGF) (Merck Millipore, GF144), and 10 ng/mL basic fibroblast growth factor (bFGF; FUJIFILM Wako, 064-04541), B27 supplement (Thermo Fisher Scientific, 17504-044), 4 μg/ml heparin (STEMCELL Technologies, 07980), 100 U/mL penicillin and 100 μg /mL streptomycin (Nacalai Tesque, 26253-84) as previously described (Tominaga, 2019). Tumor spheroids with a diameter > 75 μm were counted after 14 days for P3 cells and 10 days for HCC38 cells.

Immunocytochemistry

Patient-derived cancer cells were seeded on collagen-coated 8 chamber glass slides (Corning, 354630). One or two days later, cells were fixed with 4% paraformaldehyde /PBS (Nacalai Tesque, 09154-85) for 30 min, permeabilized in 0.1% Triton X-100 (Nacalai Tesque, 25987-85) for 10 min, and blocked with 1% BSA (Nacalai Tesque, 01863-48) for 1 h at room temperature. Then cells were incubated with the primary antibodies at optimized concentrations at 4 °C overnight. After washing with PBS, cells were incubated with secondary antibodies at appropriate dilutions at room temperature for 1 h. Nuclei were stained with 0.2 μg/mL DAPI (Dojindo, 340-07971). Slides were mounted with fluorescence mounting medium (Agilent, S3023). Images were acquired by using Zeiss LSM900 confocal microscopy and were analyzed by Image J software.

Flow Cytometry and Cell Sorting of FXYD3^{high} or FXYD3^{low} CSCs and non-CSCs

Cultured patient-derived cancer cells or cell lines were labeled by anti-FXYD3 antibody (1:100, Abcam, ab205534) at 4 °C for 30 min, and washed twice with FACS buffer. Next, these cells were incubated with Alexa Fluor 647-conjugated secondary antibody (1:1,000,

Cell Signaling Technology, #4414) at 4 °C for 30 min, and washed twice with FACS buffer. With respect to NRP1 and IGF1R, cells were labeled with PE-conjugated NRP1 antibody (R&D systems, FAB3870P) or PE-conjugated IGF1R antibody (BD Biosciences, 555999), respectively, at 4 °C for 30 min, and washed twice with FACS buffer. Cell viability solution (BD Biosciences, 555816) was used to eliminate dead cells.

Quantitative Real-time PCR

Total RNA was isolated using RNeasy Mini Kit (QIAGEN, 74106) and transcribed into cDNA using High-Capacity cDNA Reverse Transcription Kit (Thermo Fisher Scientific, 4368814), according to the manufacturer's instructions. Quantitative real-time PCR was carried out with ViiA 7 Real-Time PCR System (Thermo Fisher Scientific) using Fast SYBR Green Master Mix (Thermo Fisher Scientific, 4385612). Primers used in this experiment are as follows: *NRP1* Fwd: TACCCTGAGAATGGGTGGAC, Rev: CGTGACAAAGCGCAGAAG; *IGF1R* Fwd: TTCAGCGCTGCTGATGTG, Rev: AAGTTCCCGGCTCATGGT; *FXYD3* Fwd: GGCCAGAAGTCCGGTCA, Rev: AACGGTCCTCCACCCAATTTC (25); *FXYD3a* Fwd: AGGTTGGCGGGCTCATC, Rev: CATTTGCATTTTGCACTCATGAC (26); *FXYD3b* Fwd: TCAGCTCTCCCCAACAGGTG, Rev: AAATTGAACAAAGAGACCCTCTTGC (26);MKI67 Fwd: TGACCCTGATGAGAAAGCTCAA, Rev: CCCTGAGCAACACTGTCTTTT(27); *ATP1A1* Fwd: TGTTACTGTGGATTGGAGCG, Rev: CAACCAGTTATGATTACAACGGC; *ATP1B1* Fwd: AACCTAAGCCTCCCAAGAATG, Rev: TGCCCAGTCCAAAATACTCC; *ATP1B2* Fwd: TGGGGACTCCACCCACTAT, Rev: CATGACGAAGTTGCCGAGATT; *ATP1B3* Fwd: CCTGAAGGAGTGCCAAGGAT, Rev: GCTGTAGATACCCAACATGCAG; *GCLC* Fwd: GGCACAAGGACGTTCTCAAGT, Rev: CAGACAGGACCAACCGGAC. Housekeeping gene *ACTB* (Fwd: AAGTCCCTTGCCATCCTAAAA, Rev: ATGCTATCACCTCCCCTGTG) was used for internal control. The primer nucleotide sequences are detailed in Supplemental Table 4.

Cell Growth Assay

Cells were plated at 1,000 (patient-derived cells) or 300 (cell lines) cells/well onto collagen-coated 96-well plates in the organoid medium (for patient-derived cells) or ultra-low 96-well plates in the sphere formation medium (for cell lines). After 4 days, relative cell number was measured by CellTiter-Glo Luminescent Cell Viability Assay (Promega, G7572).

Drug Sensitivity

Cells were seeded at 1,000–2,000 cells/well onto collagen-coated 96-well plates in the organoid medium (for patient-derived cells) or ultra-low 96-well plates in the sphere formation medium (for cell lines). After 24 h, the cells were treated with paclitaxel (FUJIFILM Wako,163-

28163), doxorubicin hydrochloride (FUJIFILM Wako, 040-21521), olaparib (Selleck, AZD2281), ouabain (Sigma Aldrich, O3125), or digoxin (Sigma Aldrich, D6003) at various concentrations for 72 h. Cell viability was measured by CellTiter-Glo Luminescent Cell Viability Assay (Promega, G7572).

Combination Index

Cells were treated with drugs. Synergistic combinations were evaluated using the Chou-Talalay method, using Compusyn software (Combosyn Inc., Paramus, NJ, USA)(28). The resulting values i.e., combination index (CI), describes antagonistic (>1.0) and synergistic (<1.0) effects of the drug combinations.

Flow Cytometry Analysis after Drug Treatment

Cells were treated with drugs at a concentration of approximately IC50 for 48 h. Then cells were then incubated with anti-FXYD3, NRP1 or IGF1R antibodies and cell viability solution as described above. Analysis was carried out by flow cytometry cell sorter (BD FACSAria III). To prevent the effects of Doxorubicin fluorescence on flow cytometry data, different gating thresholds were defined for negative control (NC) and Doxorubicin-treated groups by using isotype control. The flow cytometry results were evaluated with FlowJo Software 10.7.1 (BD Biosciences).

Intracellular Ca^{2+} Detection

Intracellular Ca^{2+} was detected and quantified by flow cytometry using Oregon Green 488 BAPTA-1, AM, cell permeant (Thermo Fisher Scientific, O6807). Cells were incubated with the indicator reagent (5 μM) at 37 °C for 30 min. Then cells were washed, collected and stained with anti-FXYD3, NRP1 or IGF1R antibodies and cell viability solution for flow cytometry analysis as described above. The fluorescence signal of intracellular Ca^{2+} was quantified by geometric mean of fluorescence (GeoMFI).

ROS Detection

ROS production was detected and quantified by flow cytometry using ROS assay kit (Dojindo, R252) according to manufacturer's instructions. Briefly, cells were incubated with highly sensitive DCFH-DA Dye (1:50,000) in Hank's balanced salt solution (HBSS) at 37 °C for 30 min. After treatment, the cells were washed, collected, and then stained with anti-FXYD3, NRP1 or IGF1R antibodies and cell viability solution for flow cytometry analysis as described above. The ROS production per cell was quantified by GeoMFI.

GSH/GSSG Ratio Detection Assay

NRP1^{high}FXYD3^{high} or NRP1^{low}FXYD3^{high} CSCs were collected using anti-NRP1 and FXYD3 antibodies by FACS sorting, and were further lysed with 0.5% NP-40/PBS lysis buffer (approximately 2 ×10^5 cells/mL, Sigma Aldrich, I8896). Enzymes that may interfere with the analysis were removed using the Deproteinizing Sample Kit-TCA (Abcam, ab204708). The GSH/GSSG ratio detection was performed and calculated using GSH/GSSG Ratio Detection Assay Kit II (Abcam, ab205811), following manufacturers' instructions.

Knockdown Experiments Using siRNAs for *FXYD3*

Two different siRNAs (Thermo Fisher Scientific, #1, HSS143336, #3, HSS182369) duplexes for *FXYD3* and a nonspecific control siRNA duplex with similar GC contents (Thermo Fisher Scientific, siRNA Negative Control Med GC Duplex #2, 12935112) were transfected to cells by using Lipofectamine RNAiMAX Transfection Reagent (Thermo Fisher Scientific, 13778075), according to the manufacturers' instructions. qPCR or growth assay were performed after 48 h.

Knockdown Experiments Using Lentivirus-based System

Two independent shRNAs specific for *ATP1B1* were designed and constructed into the lentiviral vector pLKO.1-TRC cloning vector. This vector was kindly offered by Dr. David Root (Addgene plasmid # 10878; http://n2t.net/addgene:10878; RRID: Addgene_10878)(29). The sequences of shRNAs were as follows: *ATP1B1#1*, 5′–CCGGGTGATGAAGTATAACCCAAATCTCGAGATTTGGGTTATACTTCATCACTTTTTG–3′; *ATP1B1#2*, 5′–CCGGGCCGTACAGTTCACCAATCTTCTCGAGAAGATTGGTGAACTGTACGGCTTTTTG–3′. The negative control with the same sequence, which was exploited for non-mammalian shRNA control plasmid SHC002 (Sigma Aldrich), was used (shNCT). Lentivirus packaging plasmids pCMV-VSV-G-RSV-Rev and pCAG-HIVgp are kind gifts from Dr. H. Miyoshi (RIKEN, Tsukuba, Japan).

The lentivirus supernatant was produced by HEK293T cells by co-transfecting plasmids using Lipofectamine (Thermo Fisher Scientific, 18324012) and PLUS Reagent (Thermo Fisher Scientific, 11514015), as previously described(30). Next, cells were infected with virus particles for 24 h. And cells which stably express shNCT or shRNAs for *ATP1B1* were selected by adding puromycin (FUJIFILM Wako, 160-23151) (1.5 μg/mL for P3 and 0.5 μg/mL for P5) to the culture medium.

Drug Treatment *in vivo*

Dissociated patient-derived cancer cells expressing indicated shRNAs were suspended with PBS, mixed (1:1) with Matrigel (Corning, 354234), and subsequently injected into the mammary fat pads of 6–10-week-old NSG female mice at 1.5 x 10^5 P3 cells or 2 × 10^5 P5 cells (two injection sites per mouse). When the average tumor volume reached -100 mm^3, mice were randomized into control and treatment groups, and drug treatment was started. When the tumor volume of the control groups exceeded 1,000 mm^3 (P5 cells) or 2,000 mm^3 (P3 cells), mice bearing tumors were euthanized for ethical reasons. All drugs were administered by intraperitoneal injection. Treatment regimens are as follows: Vehicle-DMSO control (5% DMSO + 40% PEG 300 (FUJIFILM Wako, 164-09055) + 5% Tween 80 (TCI, T0546) + deionized-distilled H_2O [ddH_2O], twice per week), paclitaxel (20 mg/kg for P3 cells, 10 mg/kg for P5 cells, twice per week) with or without ouabain (1.5 mg/kg for P3 cells, twice per week). This dose of ouabain was selected as it is well-tolerated by aged mice (31) (Tumor growth was monitored by caliper measurements twice a week. Tumor volume was calculated as follows: $V = 4/3 \times \pi\ (S/2) \times (L/2)^2$, where S and L are the lengths of the minor and major axis, respectively. Body weights were measured every 3–4 days over treatment until endpoint.

Immunofluorescence for Tissue Sections (Frozen Tissue)

Immunofluorescence for tumor tissue sections was performed as described previously(32). Briefly, fresh tumor tissues were fixed with 4% paraformaldehyde/PBS at 4 °C overnight, and one more night incubation with 30% sucrose (FUJIFILM Wako, 196-00015)/PBS at 4 °C. Then, samples were embedded into Tissue Teck OCT compound (SAKURA, 4583) and cut into 4 μm cryosections (Leica CM1950 Cryostat). For staining, frozen tissue sections were fixed with pre-cold (−20 °C) acetone (FUJIFILM Wako, 016-00346) until evaporation. Tissue Teck OCT compound were melted at room temperature. Then, tissues were permeabilized by 0.4% Triton X-100 (Nacalai Tesque, 25987-85) at room temperature for 30 min. Next, the sections were incubated with 1% BSA blocking buffer (Nacalai Tesque, 01863-48) at room temperature for 1 h followed by incubating with primary antibodies at optimized concentrations at 4 °C overnight. On the second day, the sections were incubated with secondary antibodies conjugated with fluorescence at appropriate dilutions at room temperature for 1 h. Nuclei were counterstained with Hoechst33342 (Thermo Fisher Scientific, H3570). Images were acquired using a Zeiss LSM900 confocal microscope, and were analyzed using Image J software. The primary antibodies used in this experiment are as follows: NRP1 antibody (1:200, Thermo Fisher Scientific, 14-3042-82), IGF1R antibody (1:25, Thermo Fisher Scientific, MA5-13802), FXYD3 antibody (1:200, Abcam, ab205534), GCLC antibody (1:100, Thermo Fisher Scientific, MA5-26346).

Hematoxylin and Eosin Staining and Immunofluorescence Staining for Tissue Sections (Paraffin Block)

For paraffin sections, fresh tumor tissues were fixed with 10% formaldehyde (Nacalai Tesque, 37152-51) at room temperature overnight and embedded in paraffin. Samples were sectioned at a microtome setting of 4 μm (Thermo Fisher Scientific, HM 360 Rotary Microtome). Hematoxylin and eosin (HE) staining were performed in Kanagawa Cancer Center. For immunofluorescence staining, sections were de-paraffinized by xylene (Nacalai Tesque, 36611-03) for 10 min three times, and hydrolyzed in a graded series of ethanol (Nacalai Tesque, 14713-95). Antigen retrieval was performed by heat treatment using Target Retrieval Solution (DAKO, S2367, pH 9.0). Next, sections were incubated with Protein Block Serum-Free (DAKO, X0909) for 1 h at room temperature, followed by incubation with primary antibodies at optimized concentrations at 4 °C overnight. On the second day, the sections were incubated with secondary antibodies conjugated with fluorescence at appropriate dilutions at room temperature for 1 h. The nuclei were counterstained with 4',6-Diamidino-2-phenylindole (DAPI) (Dojindo, 340-07971). The primary antibodies used in this experiment are as follows: NRP1 antibody (1:200, Abcam, ab81321) and IGF1R antibody (1:25, Thermo Fisher Scientific, MA5-13802). A Zenon Rabbit IgG Labeling Kit (Alexa Fluor 647, Thermo Fisher Scientific, Z25308) was used for combining anti-FXYD3 antibody (final concentration 1:100, Abcam, ab205534), according to the manufacturers' instructions.

Ecocardiography

Transthoracic echocardiography and electro-cardiogram (ECG) was performed using the Vevo2100 ultrasound system (FUJIFILM VisualSonics). M-mode echocardiographic images were obtained from a short-axis view to analyze the left ventricle (LV) size and LV contraction. Echocardiographic and ECG evaluations were carried out after one week and two weeks of treatment for each mouse.

References for Supplementarl Materials and Methods

1. Tominaga K, Minato H, Murayama T, Sasahara A, Nishimura T, Kiyokawa E, et al. Semaphorin signaling via MICAL3 induces symmetric cell division to expand breast cancer stem-like cells. *Proc Natl Acad Sci U S A.* 2019;116(2):625-30.
2. Cheloni S, Hillje R, Luzi L, Pelicci PG, and Gatti E. XenoCell: classification of cellular barcodes in single cell experiments from xenograft samples. *BMC Med Genomics.* 2021;14(1):34.

3. Stuart T, Butler A, Hoffman P, Hafemeister C, Papalexi E, Mauck WM, 3rd, et al. Comprehensive Integration of Single-Cell Data. *Cell.* 2019;177(7):1888-902 e21.
4. Bach K, Pensa S, Grzelak M, Hadfield J, Adams DJ, Marioni JC, et al. Differentiation dynamics of mammary epithelial cells revealed by single-cell RNA sequencing. *Nat Commun.* 2017;8(1):2128.
5. Hänzelmann S, Castelo R, and Guinney J. GSVA: gene set variation analysis for microarray and RNA-seq data. *BMC Bioinformatics.* 2013;14:7.
6. Ritchie ME, Phipson B, Wu D, Hu Y, Law CW, Shi W, et al. limma powers differential expression analyses for RNA-sequencing and microarray studies. *Nucleic Acids Res.* 2015;43(7):e47.
7. Kim C, Gao R, Sei E, Brandt R, Hartman J, Hatschek T, et al. Chemoresistance Evolution in Triple-Negative Breast Cancer Delineated by Single-Cell Sequencing. *Cell.* 2018;173(4):879-93 e13.
8. Korsunsky I, Millard N, Fan J, Slowikowski K, Zhang F, Wei K, et al. Fast, sensitive and accurate integration of single-cell data with Harmony. *Nat Methods.* 2019;16(12):1289-96.
9. Sachs N, de Ligt J, Kopper O, Gogola E, Bounova G, Weeber F, et al. A Living Biobank of Breast Cancer Organoids Captures Disease Heterogeneity. *Cell.* 2018;172(1-2):373-86 e10.
10. Bray NL, Pimentel H, Melsted P, and Pachter L. Near-optimal probabilistic RNA-seq quantification. *Nat Biotechnol.* 2016;34(5):525-7.
11. Frankish A, Diekhans M, Ferreira AM, Johnson R, Jungreis I, Loveland J, et al. GENCODE reference annotation for the human and mouse genomes. *Nucleic Acids Res.* 2019;47(D1):D766-D73.
12. Dobin A, Davis CA, Schlesinger F, Drenkow J, Zaleski C, Jha S, et al. STAR: ultrafast universal RNA-seq aligner. *Bioinformatics.* 2013;29(1):15-21.
13. Pertea M, Pertea GM, Antonescu CM, Chang TC, Mendell JT, and Salzberg SL. StringTie enables improved reconstruction of a transcriptome from RNA-seq reads. *Nat Biotechnol.* 2015;33(3):290-5.
14. Trapnell C, Cacchiarelli D, Grimsby J, Pokharel P, Li S, Morse M, et al. The dynamics and regulators of cell fate decisions are revealed by pseudotemporal ordering of single cells. *Nat Biotechnol.* 2014;32(4):381-6.
15. La Manno G, Soldatov R, Zeisel A, Braun E, Hochgerner H, Petukhov V, et al. RNA velocity of single cells. *Nature.* 2018;560(7719):494-8.
16. Gao M, Qiao C, and Huang Y. UniTVelo: temporally unified RNA velocity reinforces single-cell trajectory inference. *Nat Commun.* 2022;13(1):6586.

17. Subramanian A, Tamayo P, Mootha VK, Mukherjee S, Ebert BL, Gillette MA, et al. Gene set enrichment analysis: a knowledge-based approach for interpreting genome-wide expression profiles. *Proc Natl Acad Sci U S A.* 2005;102(43):15545-50.
18. Cheung TH, and Rando TA. Molecular regulation of stem cell quiescence. *Nat Rev Mol Cell Biol.* 2013;14(6):329-40.
19. Gray GK, Li CM, Rosenbluth JM, Selfors LM, Girnius N, Lin JR, et al. A human breast atlas integrating single-cell proteomics and transcriptomics. *Dev Cell.* 2022;57(11):1400-20 e7.
20. Dhimolea E, de Matos Simoes R, Kansara D, Al'Khafaji A, Bouyssou J, Weng X, et al. An Embryonic Diapause-like Adaptation with Suppressed Myc Activity Enables Tumor Treatment Persistence. *Cancer Cell.* 2021;39(2):240-56 e11.
21. Macosko EZ, Basu A, Satija R, Nemesh J, Shekhar K, Goldman M, et al. Highly Parallel Genome-wide Expression Profiling of Individual Cells Using Nanoliter Droplets. *Cell.* 2015;161(5):1202-14.
22. Oki T, Nishimura K, Kitaura J, Togami K, Maehara A, Izawa K, et al. A novel cell-cycle-indicator, mVenus-p27K-, identifies quiescent cells and visualizes G0-G1 transition. *Sci Rep.* 2014;4:4012.
23. Tirosh I, Izar B, Prakadan SM, Wadsworth MH, 2nd, Treacy D, Trombetta JJ, et al. Dissecting the multicellular ecosystem of metastatic melanoma by single-cell RNA-seq. *Science.* 2016;352(6282):189-96.
24. Karaayvaz M, Cristea S, Gillespie SM, Patel AP, Mylvaganam R, Luo CC, et al. Unravelling subclonal heterogeneity and aggressive disease states in TNBC through single-cell RNA-seq. *Nat Commun.* 2018;9(1):3588.
25. Kayed H, Kleeff J, Kolb A, Ketterer K, Keleg S, Felix K, et al. FXYD3 is overexpressed in pancreatic ductal adenocarcinoma and influences pancreatic cancer cell growth. *Int J Cancer.* 2006;118(1):43-54.
26. Yamamoto H, Okumura K, Toshima S, Mukaisho K, Sugihara H, Hattori T, et al. FXYD3 protein involved in tumor cell proliferation is overproduced in human breast cancer tissues. *Biol Pharm Bull.* 2009;32(7):1148-54.
27. Sobecki M, Mrouj K, Colinge J, Gerbe F, Jay P, Krasinska L, et al. Cell-Cycle Regulation Accounts for Variability in Ki-67 Expression Levels. *Cancer Res.* 2017;77(10):2722-34.
28. Chou TC. Theoretical basis, experimental design, and computerized simulation of synergism and antagonism in drug combination studies. *Pharmacol Rev.* 2006;58(3):621-81.
29. Moffat J, Grueneberg DA, Yang X, Kim SY, Kloepfer AM, Hinkle G, et al. A lentiviral RNAi library for human and mouse genes applied to an arrayed viral high-content screen. *Cell.*

2006;124(6):1283-98.

30. Murohashi M, Hinohara K, Kuroda M, Isagawa T, Tsuji S, Kobayashi S, et al. Gene set enrichment analysis provides insight into novel signalling pathways in breast cancer stem cells. *Br J Cancer.* 2010;102(1):206-12.

31. Guerrero A, Herranz N, Sun B, Wagner V, Gallage S, Guiho R, et al. Cardiac glycosides are broad-spectrum senolytics. *Nat Metab.* 2019;1(11):1074-88.

32. Takeuchi Y, Kimura N, Murayama T, Machida Y, Iejima D, Nishimura T, et al. The membrane-linked adaptor FRS2beta fashions a cytokine-rich inflammatory microenvironment that promotes breast cancer carcinogenesis. *Proc Natl Acad Sci U S A.* 2021;118(43).

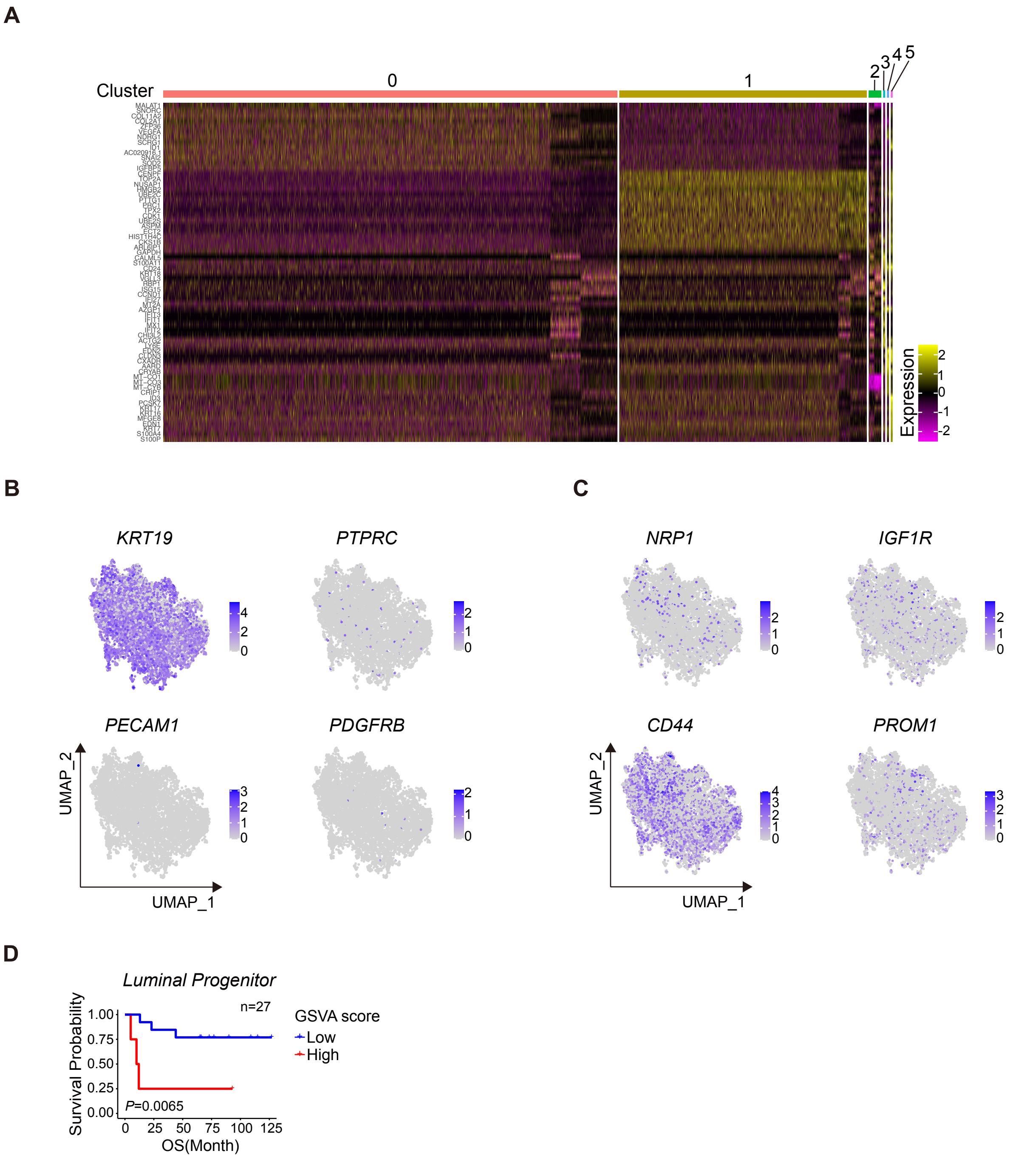

A
Cluster
0
1
2
3
4
5
Expression
2
1
0
-1
-2
B
KRT19
PTPRC
PECAM1
PDGFRB
UMAP_2
UMAP_1
C
NRP1
IGF1R
CD44
PROM1
UMAP_2
UMAP_1
D
Luminal Progenitor
n=27
GSVA score
Low
High
Survival Probability
1.00
0.75
0.50
0.25
0.00
P=0.0065
0
25
50
75
100
125
OS(Month)

Supplemental Figure1, supporting FIG.1. Tumor cells with the mammary immature traits correlate with drug resistance. (A) Heatmap showing top 10 differentially expressed genes among five clusters depicted in Figure 1B. (B and C) Uniform manifold approximation and projection (UMAP) visualization of scRNA-seq data from all the 8,390 cells in 3 PDX samples (P1, P2, P3). Expression of marker genes (Seruat, LogNormalized counts) representing cell types (B) and CSCs (C) projected onto the UMAP. (D) Kaplan–Meier survival analysis between high and low subgroups of luminal progenitor signature score by using expression profiles of TNBC tissues (n=27) in PROMIX trial. Optimal cutpoint of GSVA score was determined by the maximally selected rank statistics. P value was obtained by log-rank test. OS, overall survival.

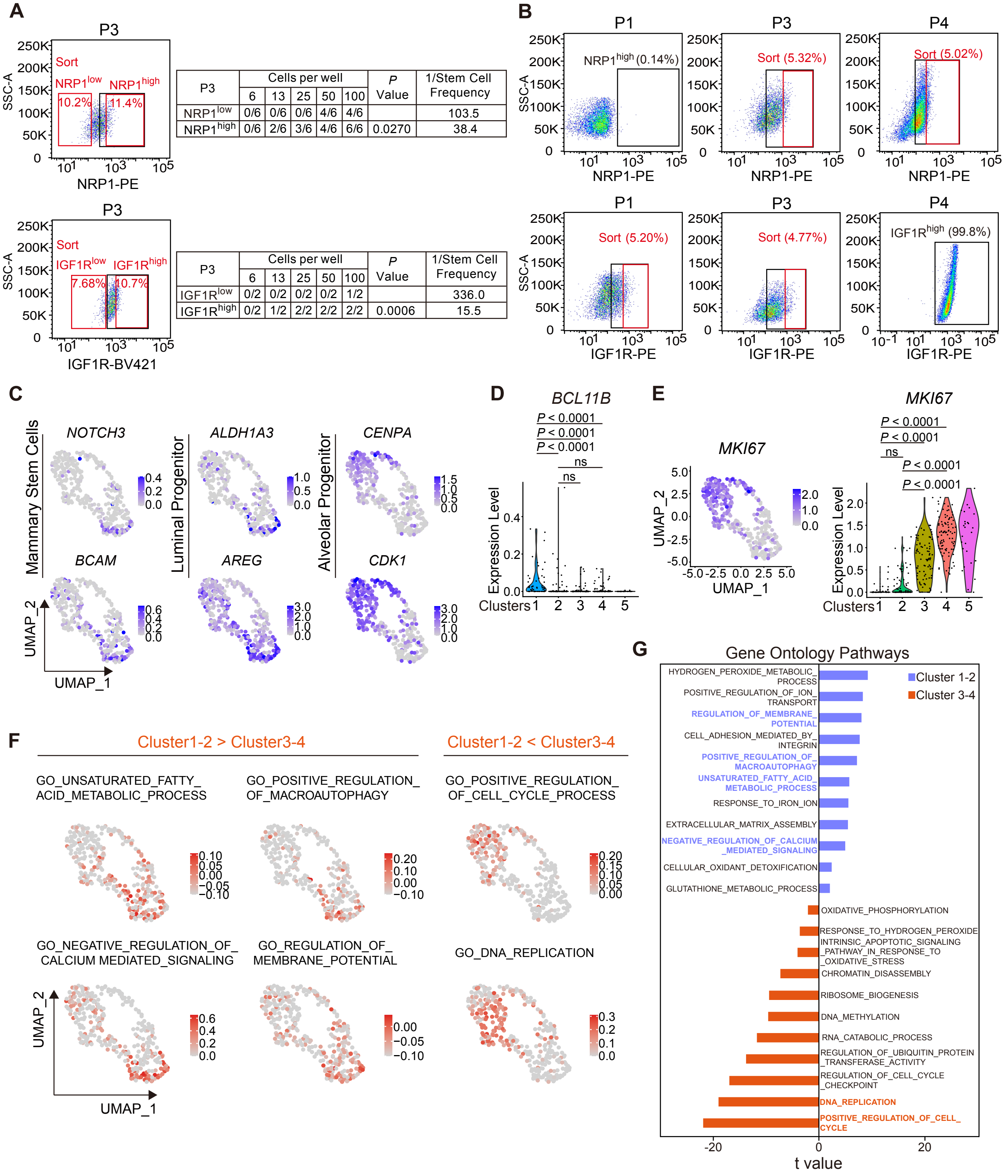

P3	Cells per well					P Value	1/Stem Cell Frequency
	6	13	25	50	100		
NRP1low	0/6	0/6	0/6	4/6	4/6		103.5
NRP1high	0/6	2/6	3/6	4/6	6/6	0.0270	38.4

P3	Cells per well					P Value	1/Stem Cell Frequency
	6	13	25	50	100		
IGF1Rlow	0/2	0/2	0/2	0/2	1/2		336.0
IGF1Rhigh	0/2	1/2	2/2	2/2	2/2	0.0006	15.5

Supplemental Figure 2, supporting FIG.2 Ancestor-like CSCs possess mammary stem or luminal progenitor-like traits and quiescence. (A) FACS sorting for the extreme limiting dilution assay (ELDA). Approximately 10 % of $NRP1^{low}$ and $NRP1^{high}$, or $IGF1R^{low}$ and $IGF1R^{high}$ cells were sorted and cultured in the ultra-low attachment 96-well plates for two weeks. (B) FACS sorting strategy for scRNA-seq to profile CSCs. Approximately top 5% of $NRP1^{high}$ or $IGF1R^{high}$ patient-derived cancer cells were sorted and subjected to analysis in the Fludigm C1 platform. $NRP1^{high}$ cells were hardly detected in the P1 sample (0.14 %). Almost all cells were $IGF1R^{high}$ cells in the P4 sample (99.8 %). These two samples were not used for analysis. (C) Expression levels (Seruat, LogNormalized counts) of representative genes of mammary gland stem/progenitors in the UMAP visualization. (D) Violin plots of expression (Seruat, LogNormalized counts) of BCL11B in each cluster shown in Figure 2C. (E) Expression levels (Seruat, LogNormalized counts) of MKI67 in the UMAP visualization (left) and Violin plots (right). Statistical significance was determined by Kruskal-Wallis test with Dunn's multiple comparisons test. ns, not significant. (F) UMAP visualization of representative enriched Gene Ontology (GO) pathways with GSVA score. (G) Representative differentially enriched gene ontology (GO) pathways between cluster 1–2 and cluster 3–4 using gene set variance analysis (GSVA) ($Padj < 0.05$).

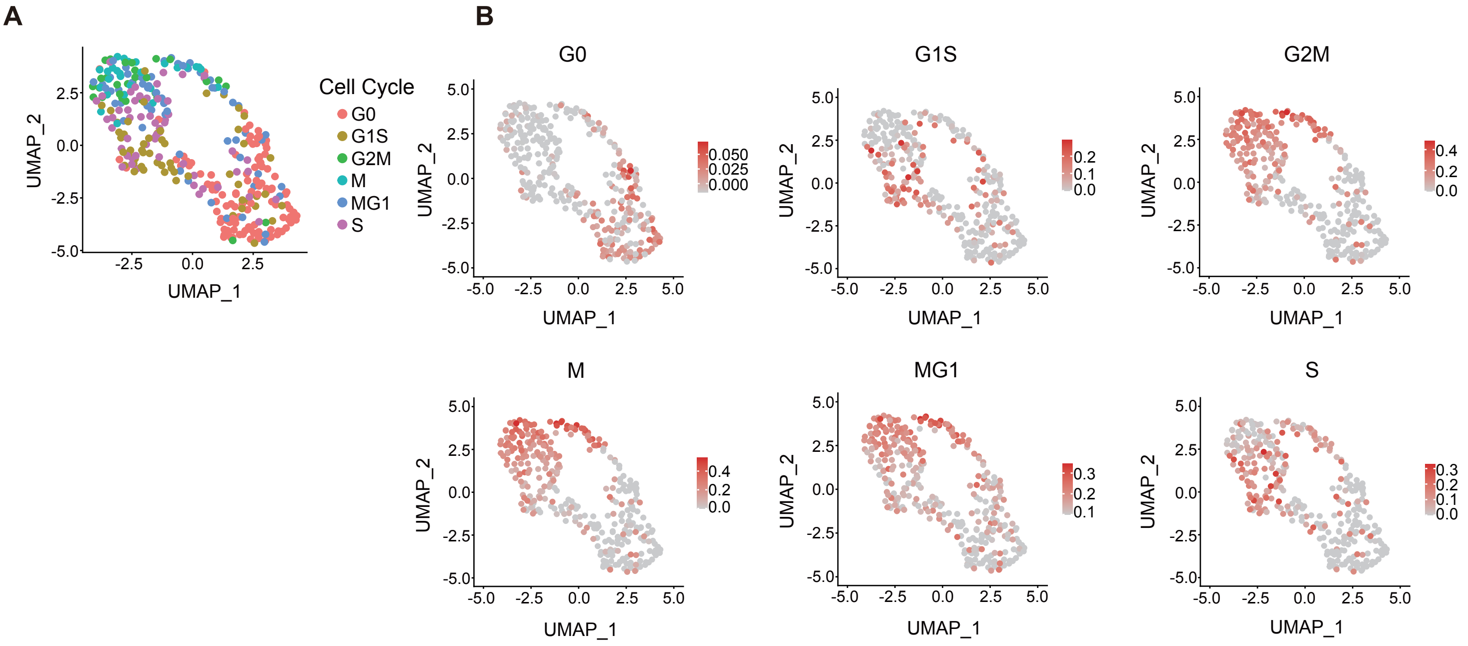

A
B
Cell Cycle
G0
G1S
G2M
M
MG1
S
UMAP_1
UMAP_2
G0
G1S
G2M
M
MG1
S

Supplemental Figure 3, supporting FIG.2 Ancestor-like CSCs possess mammary stem or luminal progenitor-like traits and quiescence. (A) UMAP visualization of SMART-seq data used in Figure 2C and D. Cells were colored by cell cycle stages. (B) Z-score of gene signatures of each cell cycle stage.

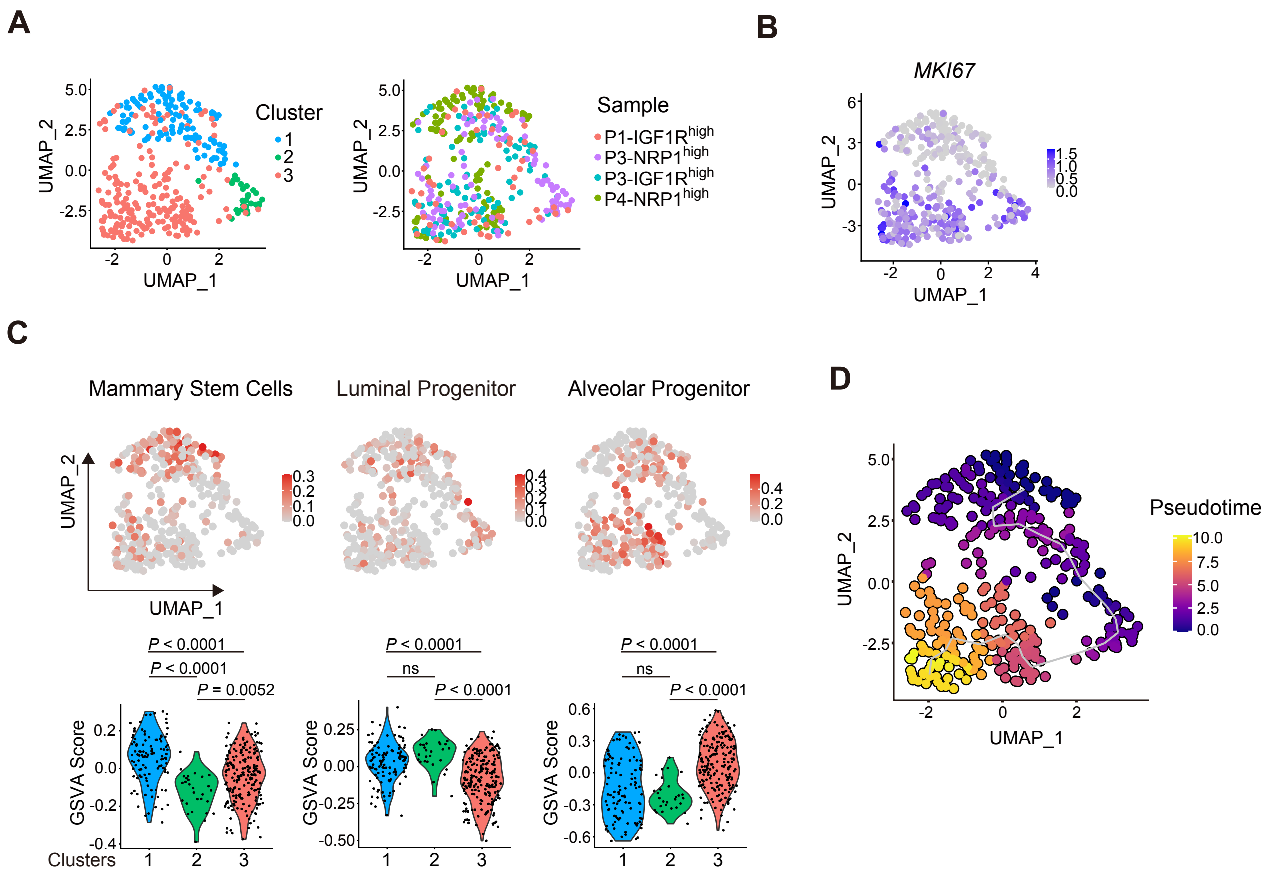

A
UMAP_2
UMAP_1
Cluster
1
2
3
Sample
P1-IGF1Rhigh
P3-NRP1high
P3-IGF1Rhigh
P4-NRP1high
B
MKI67
C
Mammary Stem Cells
Luminal Progenitor
Alveolar Progenitor
P < 0.0001
P = 0.0052
ns
GSVA Score
Clusters
D
Pseudotime

Supplemental Figure 4, supporting FIG.2. Ancestor-like CSCs possess mammary stem or luminal progenitor-like traits and quiescence without affecting by cell cycle-related genes. (A) UMAP visualization of SMART-seq data after removing the effects of cell cycle regulation from all the cells in four cell populations (IGF1R^{high} cells in P1, NRP1^{high} cells in P3, IGF1R^{high} cells in P3, and NRP1^{high} cells in P4), colored by their unsupervised clusters (left) and samples (right). (B) Expression levels (Seruat, LogNormalized counts) of MKI67 in the UMAP visualization. (C) Top, GSVA score of gene signatures of mammary gland stem/progenitors. Bottom, Violin plots of GSVA score for each cluster. Statistical significance was determined by one-way ANOVA with Bonferroni post hoc test. ns, not significant. (D) UMAP visualization of SMART-seq data from the cells colored by pseudotime.

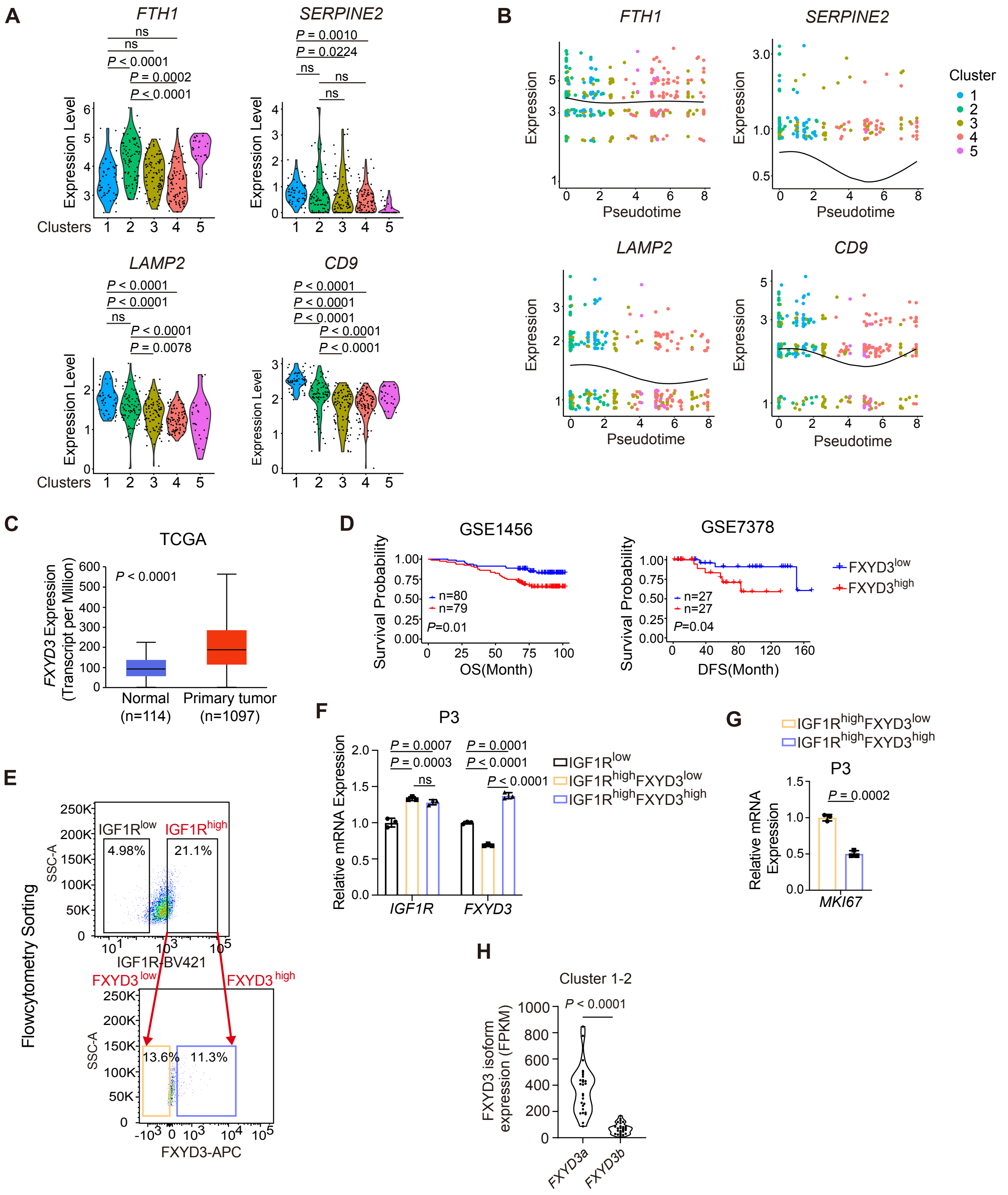
A
FTH1
SERPINE2
LAMP2
CD9
ns
P < 0.0001
P = 0.0002
P = 0.0010
P = 0.0224
P = 0.0078
Expression Level
Clusters
B
Expression
Pseudotime
Cluster
C
TCGA
FXYD3 Expression (Transcript per Million)
P < 0.0001
Normal (n=114)
Primary tumor (n=1097)
D
GSE1456
GSE7378
Survival Probability
n=80
n=79
P=0.01
n=27
n=27
P=0.04
OS(Month)
DFS(Month)
FXYD3low
FXYD3high
E
Flowcytometry Sorting
IGF1Rlow
IGF1Rhigh
4.98%
21.1%
SSC-A
IGF1R-BV421
FXYD3 low
FXYD3 high
13.6%
11.3%
FXYD3-APC
F
P3
Relative mRNA Expression
P = 0.0007
P = 0.0003
P = 0.0001
P < 0.0001
IGF1Rlow
IGF1RhighFXYD3low
IGF1RhighFXYD3high
IGF1R
FXYD3
G
P = 0.0002
Relative mRNA Expression
MKI67
H
Cluster 1-2
FXYD3 isoform expression (FPKM)
FXYD3a
FXYD3b

Supplemental Figure 5, supporting FIG.3. Plasma membrane FXYD3 demarcates ancestor-like CSCs. (A) Violin plots of expression (Seruat, Log Normalized counts) of each gene in each cluster. Statistical significance was determined by Kruskal-Wallis test with Dunn's multiple comparisons test. (B) Changes in expression (Seruat, LogNormalized counts) of each gene during pseudotime. (C) Box plot showing the expression of FXYD3 between primary tumor tissues and normal tissues in TCGA breast invasive carcinoma dataset. Statistical significance was determined with unpaired two-tailed Student's t tests. (D) Kaplan–Meier survival analysis between high and low expression of FXYD3 in primary breast tumor tissues of GSE1456 (n = 159) and GSE7378 (n = 54) datasets. Medians were used as cut-off value. P-value was obtained by log-rank test. DFS; disease-free survival. (E) FACS sorting strategy using the combination of IGF1R and FXYD3 antibodies. (F and G) Relative mRNA expression levels of IGF1R, FXYD3 (F) and MKI67 (G) measured by qPCR between IGF1R^{low}, IGF1R^{high}FXYD3^{low}, and IGF1R^{high}FXYD3^{high} cells. Values were normalized to ACTB and fold changes were calculated relative to the values of IGF1R^{low} (F) or IGF1R^{high}FXYD3^{low} (G) cells. (H) FXYD3 isoform expression in clusters 1 and 2 in P3 cells. (F) Statistical significance was determined by one-way ANOVA with Bonferroni post hoc test.(G and H) Statistical significance was determined by unpaired two-tailed Student's t-tests. Results are shown as means ± SD. n = 3.

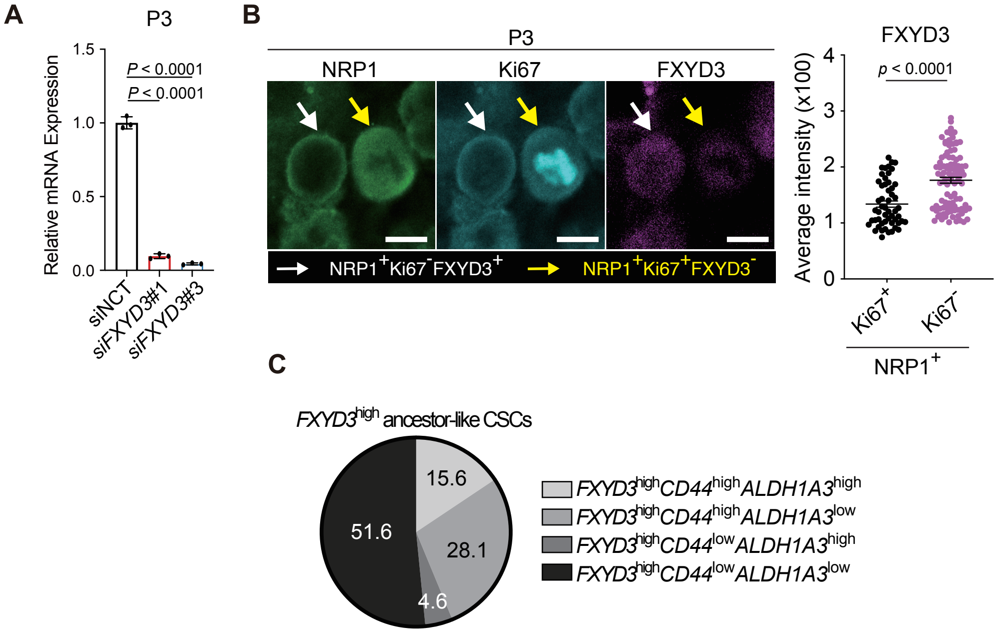

A
P3
Relative mRNA Expression
1.5
1.0
0.5
0.0
P < 0.0001
P < 0.0001
siNCT
siFXYD3#1
siFXYD3#3
B
P3
NRP1
Ki67
FXYD3
NRP1+Ki67-FXYD3+
NRP1+Ki67+FXYD3-
FXYD3
p < 0.0001
Average intensity (x100)
0
1
2
3
4
Ki67+
Ki67-
NRP1+
C
FXYD3high ancestor-like CSCs
15.6
28.1
4.6
51.6
FXYD3highCD44highALDH1A3high
FXYD3highCD44highALDH1A3low
FXYD3highCD44lowALDH1A3high
FXYD3highCD44lowALDH1A3low

Supplemental Figure 6, supporting FIG.4. FXYD3 expression demarcates ancestor-like CSCs. (A) Cells were transfected with siRNAs for FXYD3 or control siRNAs (siNCT) and relative mRNA expression levels of FXYD3 were measured by qPCR after 48 h. Values were normalized to ACTB and fold changes were calculated relative to the values of control cells (siNCT). Statistical significance was determined by one-way ANOVA with Dunnett's multiple comparisons test. n = 3. (B) Left, Immunofluorescence staining of P3 cells using antibodies against NRP1, FXYD3 or Ki67. White arrows indicate the cell positive for NRP1 and FXYD3 but negative for Ki67. Yellow arrows indicate the cell positive for NRP1 and Ki67 but negative for FXYD3. Scale bars: 20 μm. Right, Quantification of average intensities of FXYD3 staining in each cell in $NRP1^{+}Ki67^{+}$ cells and $NRP1^{+}Ki67^{-}$ cells. Statistical significance was determined by unpaired, two-tailed Student's t-test. Results are shown as means ± SEM. n = 53 cells for $NRP1^{+}Ki67^{+}$ cells and n = 95 cells for $NRP1^{+}Ki67^{-}$ cells were counted in 40 random fields. (C) Cells with top 25% highest expression levels of each gene derived from C1 RNA-seq data were quantified. Cells were grouped into $FXYD3^{high}CD44^{high}ALDH1A3^{high}$, $FXYD3^{high}CD44^{high}ALDH1A3^{low}$, $FXYD3^{high}CD44^{low}ALDH1A3^{high}$ and $FXYD3^{high}CD44^{low}ALDH1A3^{low}$ population and proportion of each group (%) were shown in pie chart.

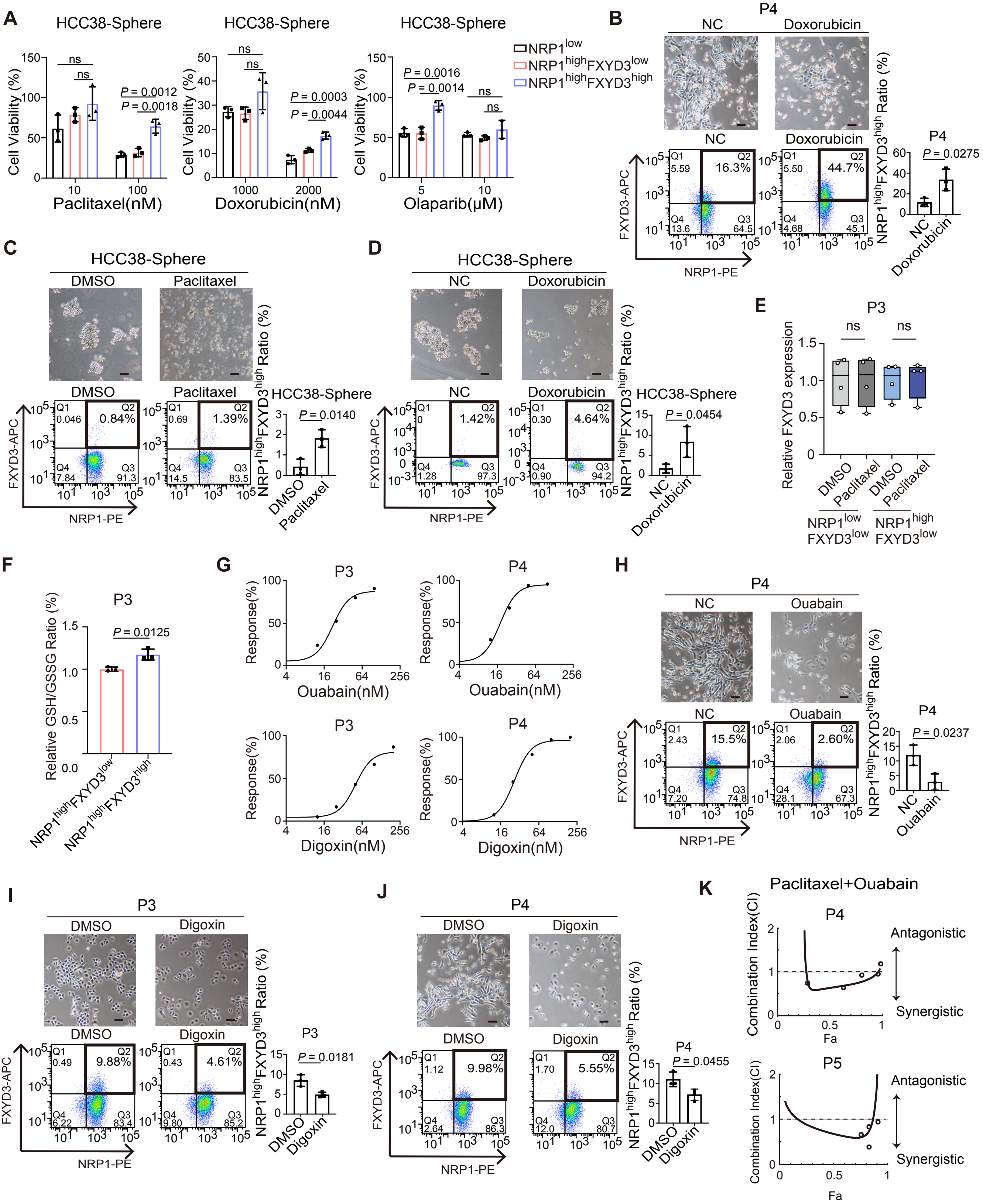
A
HCC38-Sphere
Cell Viability (%)
Paclitaxel(nM)
P = 0.0012
P = 0.0018
ns
Doxorubicin(nM)
P = 0.0003
P = 0.0044
Olaparib(μM)
P = 0.0016
P = 0.0014
NRP1^low
NRP1^high FXYD3^low
NRP1^high FXYD3^high
B
P4
NC
Doxorubicin
FXYD3-APC
NRP1-PE
16.3%
44.7%
NRP1^high FXYD3^high Ratio (%)
P = 0.0275
C
HCC38-Sphere
DMSO
Paclitaxel
0.84%
1.39%
P = 0.0140
D
NC
Doxorubicin
1.42%
4.64%
P = 0.0454
E
P3
Relative FXYD3 expression
NRP1^low FXYD3^low
NRP1^high FXYD3^low
F
P3
Relative GSH/GSSG Ratio (%)
P = 0.0125
NRP1^high FXYD3^low
NRP1^high FXYD3^high
G
P3
P4
Response(%)
Ouabain(nM)
Digoxin(nM)
H
P4
NC
Ouabain
15.5%
2.60%
P = 0.0237
I
P3
DMSO
Digoxin
9.88%
4.61%
P = 0.0181
J
P4
DMSO
Digoxin
9.98%
5.55%
P = 0.0455
K
Paclitaxel+Ouabain
P4
P5
Combination Index(CI)
Fa
Antagonistic
Synergistic

Supplemental Figure 7, supporting FIG.5. Na^+/K^+ pump inhibition decreases $FXYD3^{high}$ ancestor-like CSCs proportions and sensitizes them to drugs. (A) drug sensitivity assays. Statistical significance was determined by one-way ANOVA with Bonferroni post hoc test. n = 3. (B) P4 patient-derived cancer cells after 48 h doxorubicin (0.25 μM) treatment. Scale bars: 100 μm. FACS analysis (bottom left). The ratio (%) of $NRP1^{high}FXYD3^{high}$ cells to total cells were quantitated based on FACS analysis (bottom right). n = 3. (C and D) Top, HCC38-spheroids after 48 h paclitaxel (0.5 μM) (C) or doxorubicin (1.0 μM) (D) treatment. Scale bars: 100 μm. FACS analysis showed the enrichment of $NRP1^{high}FXYD3^{high}$ cells after drug treatment (left bottom). The ratio (%) of $NRP1^{high}FXYD3^{high}$ cells to total cells were quantitated based on FACS analysis (right bottom). n = 3. (E) FXYD3 expression levels were measured by FACS analysis. $NRP1^{high}FXYD3^{low}$ or $NRP1^{low}FXYD3^{low}$ cells were treated with 10 nM paclitaxel for 12 h. n = 4. ns, not significant. (F) After FACS sorting of P3 patient-derived cancer cells with antibodies against NRP1 and FXYD3, the ratio (%) of GSH/GSSG was measured. n = 3. (G) Dose-response curves of P3 and P4 cells treated with ouabain or digoxin for 72 h. (H) Top, P4 cells after treatment with Na^+/K^+ pump inhibitor ouabain (50 nM) or vehicle-alone (negative control [NC]). Scale bars: 100 μm. FACS analysis (bottom left). The ratio (%) of $NRP1^{high}FXYD3^{high}$ cells to total cells were quantitated based on FACS analysis (bottom right). n = 3. (I and J Top, P3 (I) or P4 (J) cells after 48 h treatment of Na^+/K^+ pump inhibitor digoxin (50 nM). Scale bars: 100 μm. Representative FACS analysis showed the decrease in $NRP1^{high}FXYD3^{high}$ cells after digoxin treatment (left bottom). The ratio (%) of $NRP1^{high}FXYD3^{high}$ cells to total cells were quantitated based on FACS analysis (right bottom). n = 3. (K) Combined effects of paclitaxel and ouabain. Combination Index (CI) > 1 and < 1 indicates antagonistic and synergistic effects, respectively. B, C, D, E, F, H, I and J, Statistical significance was determined with unpaired two-tailed Student's t-tests. Results are shown as means ± SD.

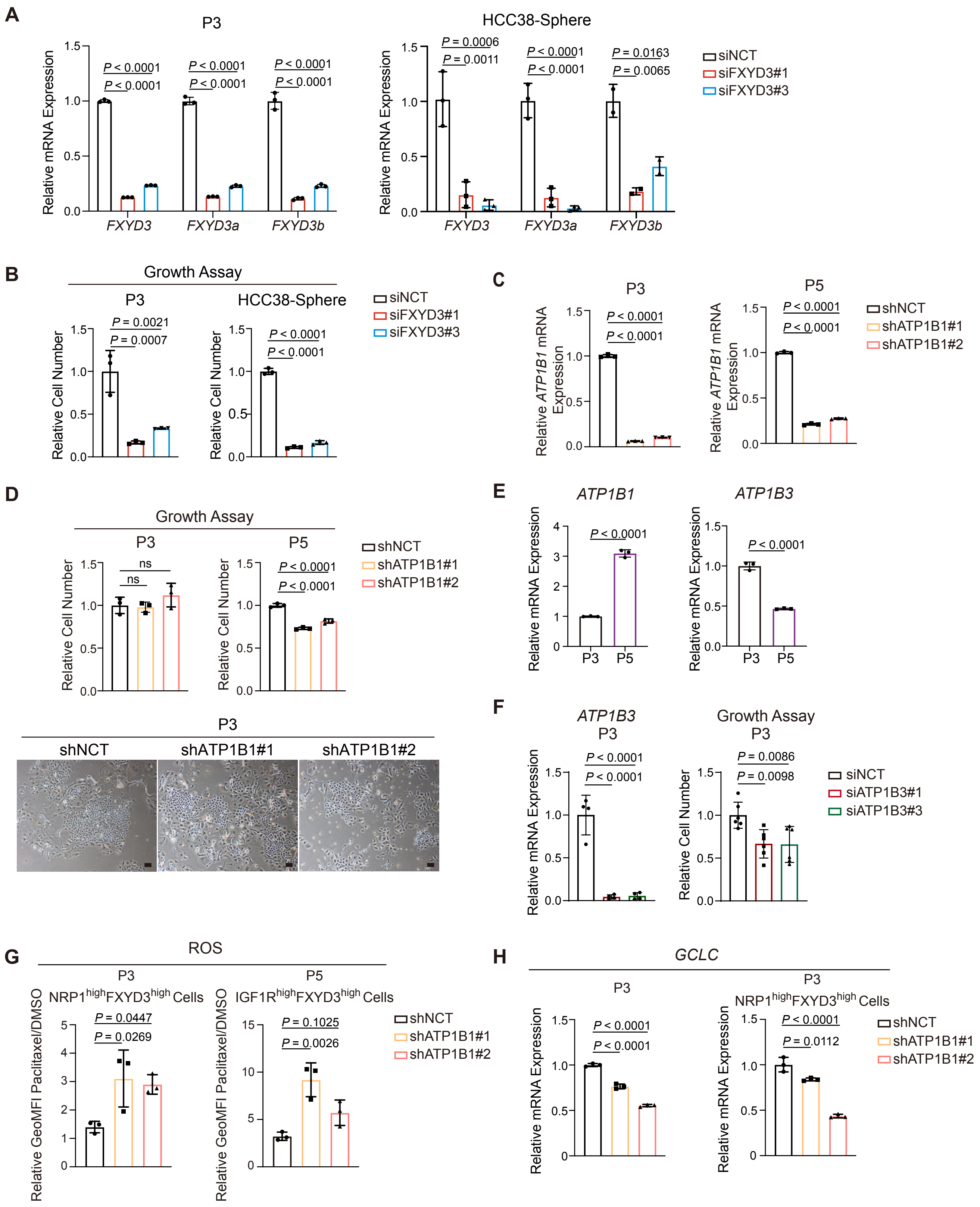

A
P3
HCC38-Sphere
Relative mRNA Expression
P < 0.0001
P = 0.0006
P = 0.0011
P = 0.0163
P = 0.0065
FXYD3
FXYD3a
FXYD3b
siNCT
siFXYD3#1
siFXYD3#3
B
Growth Assay
Relative Cell Number
P = 0.0021
P = 0.0007
C
Relative ATP1B1 mRNA Expression
P5
shNCT
shATP1B1#1
shATP1B1#2
D
ns
E
ATP1B1
ATP1B3
F
siATP1B3#1
siATP1B3#3
P = 0.0086
P = 0.0098
G
ROS
NRP1^high FXYD3^high Cells
IGF1R^high FXYD3^high Cells
Relative GeoMFI Paclitaxel/DMSO
P = 0.0447
P = 0.0269
P = 0.1025
P = 0.0026
H
GCLC
P = 0.0112

Supplemental Figure 8, supporting FIG.6. Na^+/K^+ pump inhibition decreases FXYD3^{high} ancestor-like CSCs proportions and sensitize them to drugs. (A) Cells were transfected with siRNAs for FXYD3 or control siRNAs (siNCT) and relative mRNA expression levels of FXYD3, FXYD3a, FXYD3b were measured by qPCR after 48 h. Values were normalized to ACTB and fold changes were calculated relative to the values of control cells (siNCT). n = 3. (B) Cell growth assay was performed after transfected with siRNAs for FXYD3 (siFXYD3#1 and siFXYD3#3) or negative control siRNAs (siNCT). n = 3. (C) Relative mRNA expression levels of ATP1B1 in P3 and P5 patient-derived cancer cells transduced with shRNAs for ATP1B1 (shATP1B1#1 and shATP1B1#2) or negative control shRNA (shNCT) measured by qPCR. Values were normalized to ACTB and fold changes calculated relative to the values of control cells (shNCT). n = 3. (D) Cell growth assay after transduced with shRNAs for ATP1B1 or negative control shRNA (shNCT) in P3 or P5 cells (top). n = 3. Representative images of P3 cells after transduced with shRNAs for ATP1B1 or negative control shRNA (shNCT) (bottom). (E) Relative mRNA expression levels of ATP1B1, ATP1B2, ATP1B3 were measured by qPCR. Values were normalized to ACTB and fold changes were calculated relative to the values of P3 cells. Statistical significance was determined with unpaired two-tailed Student's t-tests. n = 3. (F) Relative mRNA expression levels of ATP1B3 in P3 patient-derived cancer cells transfected with siRNAs for ATP1B3 or negative control siRNA (siNCT) measured by qPCR (left). n = 4. Cell growth assay was performed after transfected with siRNAs for ATP1B3 or negative control siRNA (siNCT) in P3 cells (right). n = 4. (G) After knockdown of ATP1B1, GeoMFI of cellular ROS levels were measured by FACS. n = 3. (H) After knockdown of ATP1B1, relative mRNA expression levels of GCLC were measured by qPCR (left). After knockdown of ATP1B1, NRP1^{high}FXYD3^{high} cells were sorted by FACS and the relative mRNA expression levels of GCLC were measured by qPCR (right). Values were normalized to ACTB and fold changes were calculated relative to the values of control cells (shNCT). n = 3. A, B, C, D, F, G and H, Statistical significance was determined by one-way ANOVA with Dunnett's multiple comparisons test. Results are shown as means ± SD. ns, not significant.

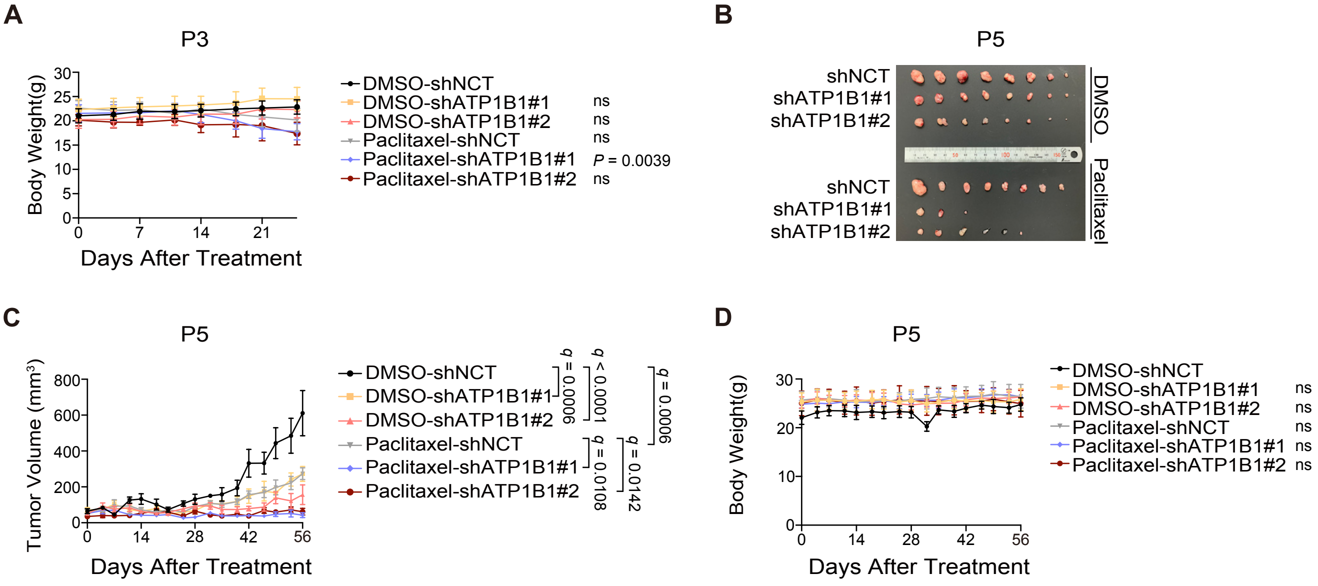
A
P3
Body Weight(g)
Days After Treatment
DMSO-shNCT
DMSO-shATP1B1#1 ns
DMSO-shATP1B1#2 ns
Paclitaxel-shNCT ns
Paclitaxel-shATP1B1#1 P = 0.0039
Paclitaxel-shATP1B1#2 ns
B
P5
shNCT
shATP1B1#1
shATP1B1#2
DMSO
shNCT
shATP1B1#1
shATP1B1#2
Paclitaxel
C
P5
Tumor Volume (mm3)
Days After Treatment
DMSO-shNCT
DMSO-shATP1B1#1
DMSO-shATP1B1#2
Paclitaxel-shNCT
Paclitaxel-shATP1B1#1
Paclitaxel-shATP1B1#2
q = 0.0006
q < 0.0001
q = 0.0006
q = 0.0108
q = 0.0142
D
P5
Body Weight(g)
Days After Treatment
DMSO-shNCT
DMSO-shATP1B1#1 ns
DMSO-shATP1B1#2 ns
Paclitaxel-shNCT ns
Paclitaxel-shATP1B1#1 ns
Paclitaxel-shATP1B1#2 ns

Supplemental Figure 9, supporting FIG.7. Knockdown of ATP1B1 sensitized TNBC PDX tumors to paclitaxel treatment and decreased proportion of FXYD3^{high} ancestor-like CSCs. (A, D) Body weight curves after drug treatment. n = 4 mice (A and D) for each condition. Body weight at end point was compared with DMSO-shNCT (A and D). Combo, a combination of paclitaxel and ouabain. Statistical significance was determined with unpaired two-tailed Student's t-tests. (B) Images of tumors generated in mice. (C) Tumor growth curves during paclitaxel treatment. n = 6 for each condition of P5 PDX. Statistical significance was determined by two-way ANOVA with two-stage linear step-up procedure of Benjamini, Krieger and Yekutieli post hoc tests. Results are shown as means ± SEM.

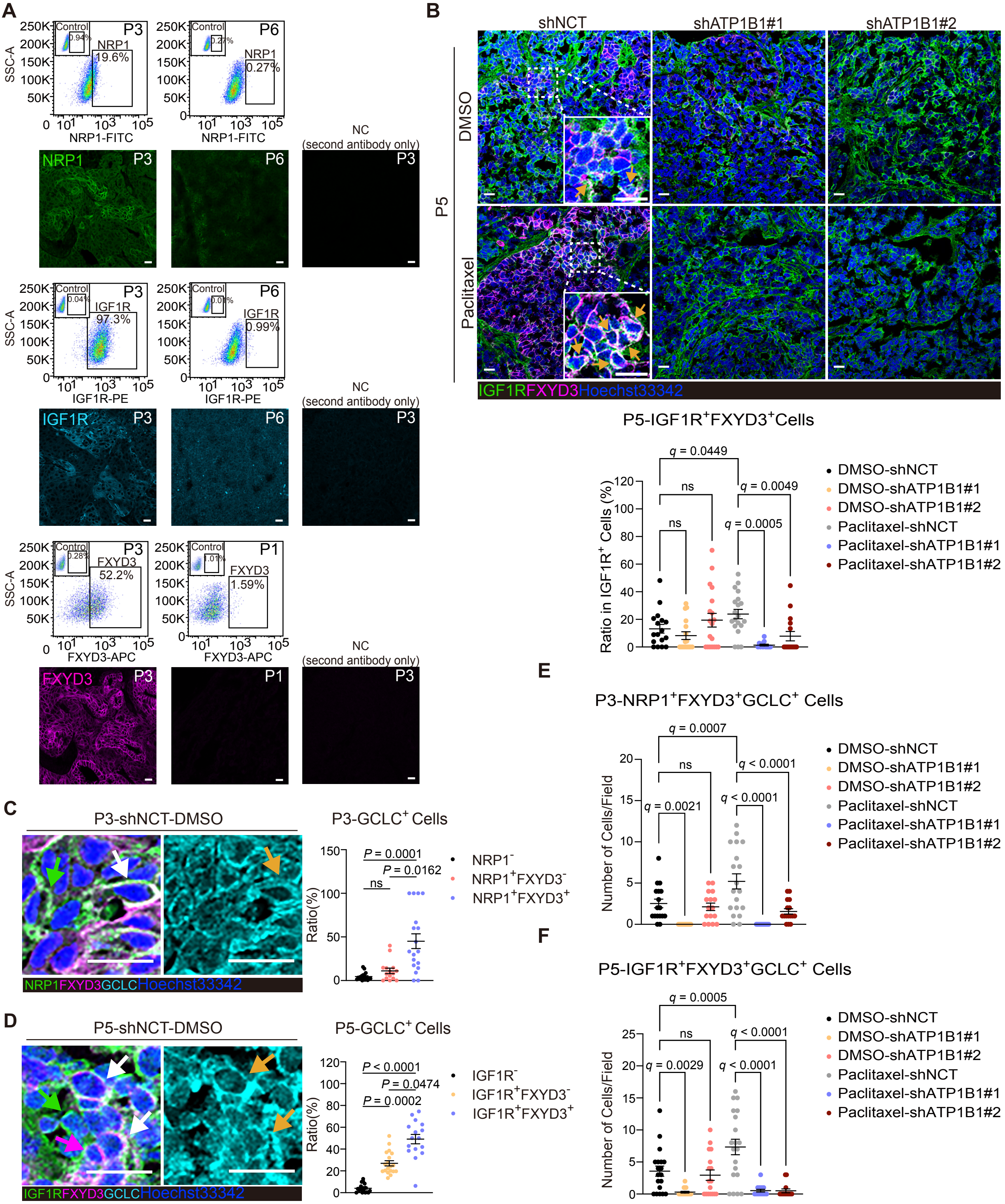
A
B
C
D
E
F
P5-IGF1R+FXYD3+Cells
P3-NRP1+FXYD3+GCLC+ Cells
P5-IGF1R+FXYD3+GCLC+ Cells
P3-GCLC+ Cells
P5-GCLC+ Cells
P3-shNCT-DMSO
P5-shNCT-DMSO
shNCT
shATP1B1#1
shATP1B1#2
DMSO
Paclitaxel
IGF1R FXYD3 Hoechst33342
NRP1 FXYD3 GCLC Hoechst33342
IGF1R FXYD3 GCLC Hoechst33342
NC (second antibody only)
DMSO-shNCT
DMSO-shATP1B1#1
DMSO-shATP1B1#2
Paclitaxel-shNCT
Paclitaxel-shATP1B1#1
Paclitaxel-shATP1B1#2

Supplemental Figure 10, supporting FIG.7. Knockdown of ATP1B1 sensitized TNBC PDX tumors to paclitaxel treatment and decreased proportion of FXYD3-positive ancestor-like CSCs. (A) FACS analysis results with each antibody or isotype control antibody, and representative images of immunofluorescence staining using each antibody. Ratio (%) of cells expressing moderate to high levels of each protein to total cells are shown. As a comparison, ratio (%) of cells bound to control antibodies at similar levels are shown (inset). P3 cells stained only with secondary antibodies were shown as negative control (NC). Scale bars: 20 μm. (B) Top, Immunofluorescence staining of frozen tissues of PDX tumors using antibodies against IGF1R and FXYD3 and the nuclei were stained using Hoechst33342. Yellow arrows indicate the cells double positive for IGF1R and FXYD3. Scale bars: 20 μm. Bottom, Quantification of the percentage of IGF1R and FXYD3-double positive cells to total IGF1R-positive cells. n = 16–20 random fields were collected for each condition. (C and D) Left, Representative images of immunofluorescence staining of frozen sections of PDX tumors derived from control DMSO-treated cells, using antibodies against NRP1, FXYD3 and GCLC (C) or IGF1R, FXYD3 and GCLC (D) and the nuclei were stained by Hoechst33342. White arrows indicate the cells double positive for NRP1 and FXYD3 (C) or the cells double positive for IGF1R and FXYD3 (D). Yellow arrows indicate the cells triple positive for NRP1, FXYD3 and GCLC (C) or IGF1R, FXYD3 and GCLC (D). Scale bars: 20 μm. Right, Quantification of the ratio (%) of GCLC-positive cells to each cell population. n = 18-20 random fields were collected for each condition. (E and F) Quantification of the number of the cells triple positive for NRP1, FXYD3 and GCLC (E) or the number of the cells triple positive for IGF1R, FXYD3 and GCLC (F) in each condition. n = 16-20 random fields were collected for each condition. B, C, D, E and F, Outliers were excluded with ROUT method before statistical analysis. B, E and F, Statistical significance was determined by two-way ANOVA with two-stage linear step-up procedure of Benjamini, Krieger and Yekutieli post hoc tests. C and D, Kruskal-Wallis test with Dunn's multiple comparisons test. Results are shown as means ± SEM.

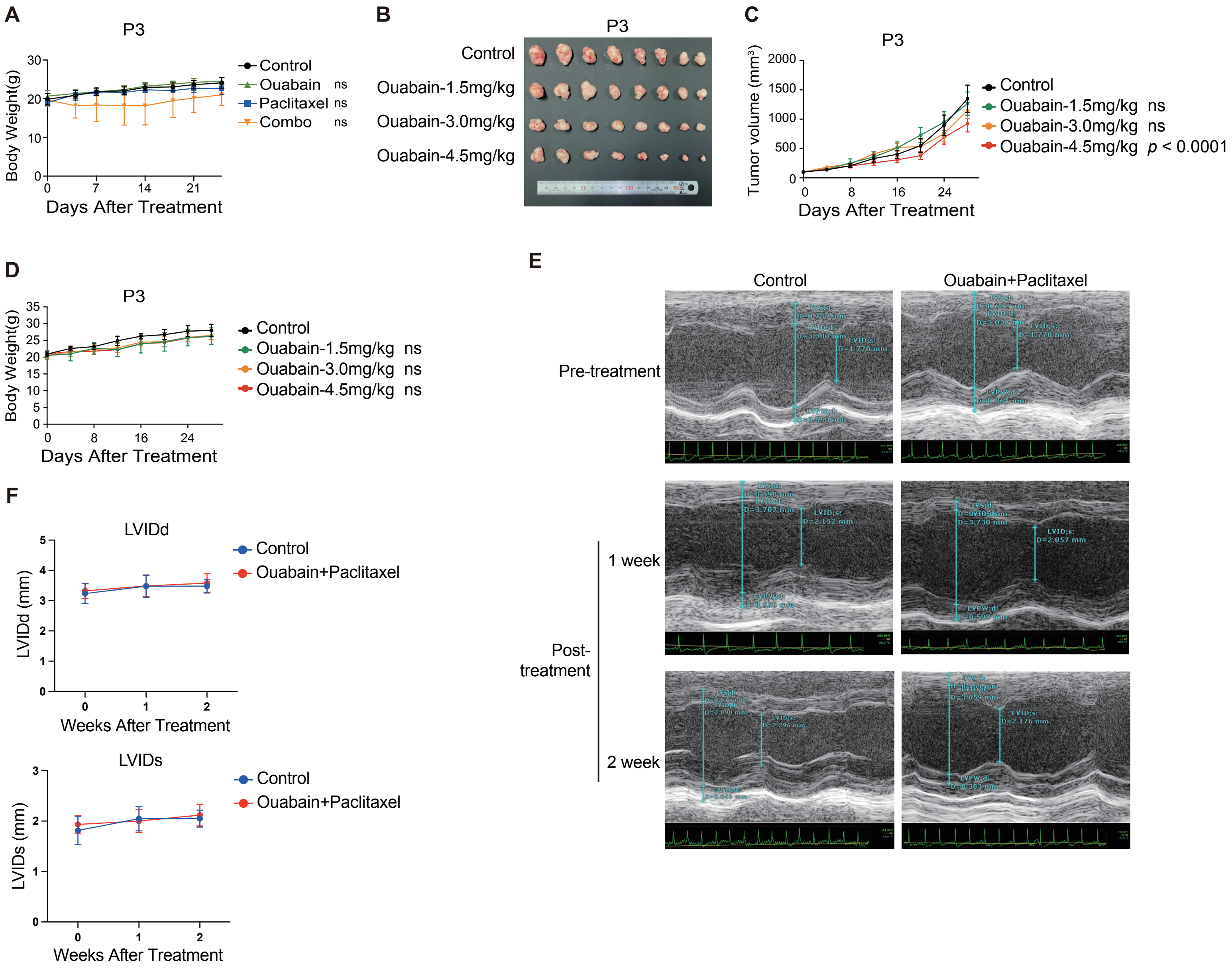

A
P3
Body Weight(g)
Days After Treatment
Control
Ouabain ns
Paclitaxel ns
Combo ns
B
P3
Control
Ouabain-1.5mg/kg
Ouabain-3.0mg/kg
Ouabain-4.5mg/kg
C
P3
Tumor volume (mm³)
Days After Treatment
Control
Ouabain-1.5mg/kg ns
Ouabain-3.0mg/kg ns
Ouabain-4.5mg/kg p < 0.0001
D
P3
Body Weight(g)
Days After Treatment
Control
Ouabain-1.5mg/kg ns
Ouabain-3.0mg/kg ns
Ouabain-4.5mg/kg ns
E
Control
Ouabain+Paclitaxel
Pre-treatment
Post-treatment
1 week
2 week
F
LVIDd
LVIDd (mm)
Weeks After Treatment
Control
Ouabain+Paclitaxel
LVIDs
LVIDs (mm)
Weeks After Treatment
Control
Ouabain+Paclitaxel

Supplemental Figure 11, supporting FIG.7. Ouabain treatment inhibits tumorigenesis and treatment with ouabain and paclitaxel shows lack of cardiotoxicity. (A) Body weight curves after drug treatment. n = 5 mice for each condition. Body weight at end point was compared with Control. (B) Images of tumors generated in mice. (C) Tumor growth curves during ouabain treatment. n = 4 for each condition of P3 PDX. Statistical significance was determined by two-way ANOVA with two-stage linear step-up procedure of Benjamini, Krieger and Yekutieli post hoc tests. Results are shown as means ± SEM. (D) Body weight curves after drug treatment. n = 4 for each condition of P3 PDX. (E) M-mode echocardiographic images of pre- and post-treatment of ouabain and paclitaxel. (F) Quantification of the left ventricle internal dimension systolic phase (LVIDs) and LVID diastolic phase (LVIDd) by transthoratic echocardiography. n = 7 mice for each condition. Statistical significance was determined by unpaired, two-tailed Student's t-tests. Results are shown as mean ± SEM.

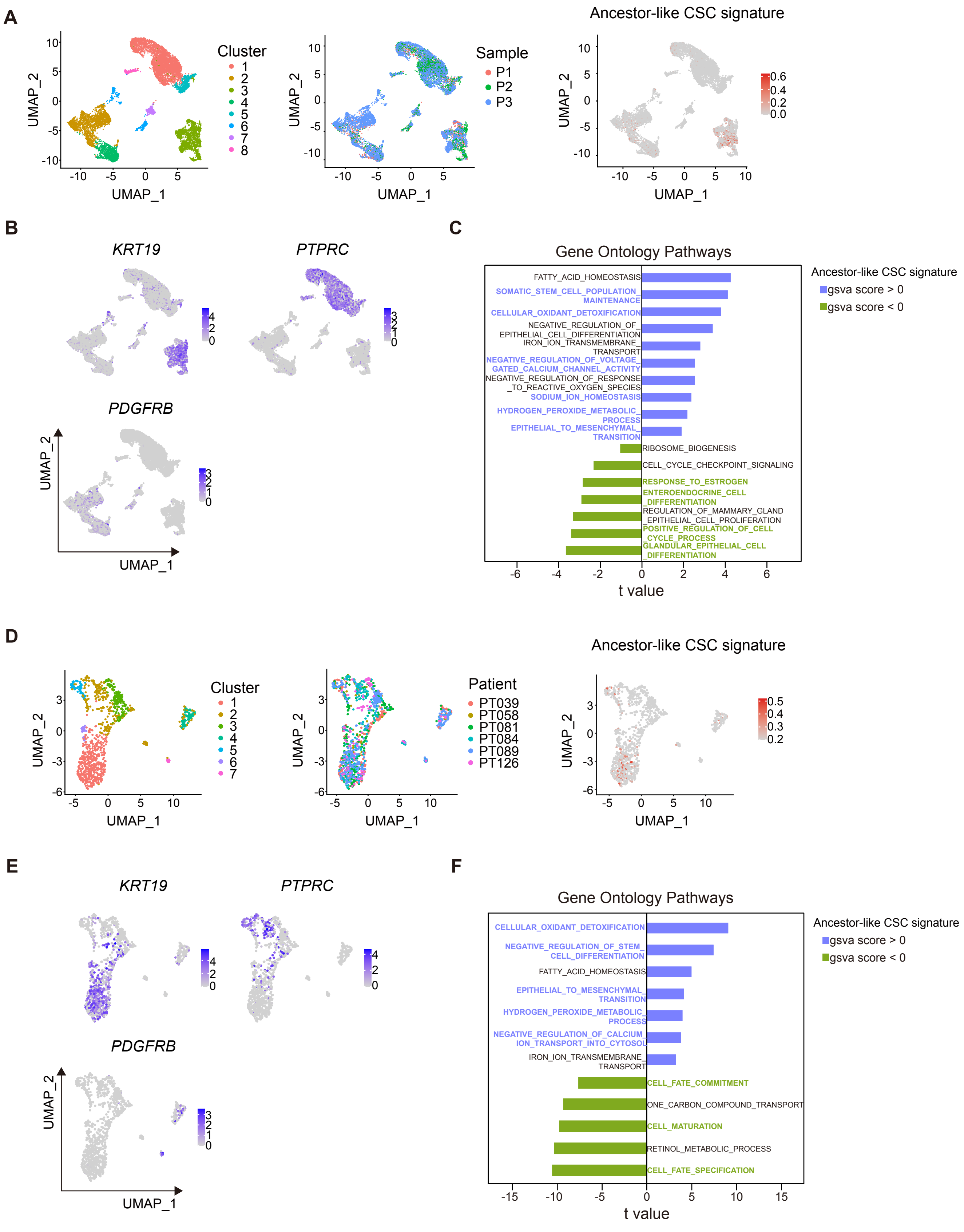
A
Ancestor-like CSC signature
Cluster
Sample
P1
P2
P3
UMAP_1
UMAP_2
B
KRT19
PTPRC
PDGFRB
C
Gene Ontology Pathways
Ancestor-like CSC signature
gsva score > 0
gsva score < 0
FATTY_ACID_HOMEOSTASIS
SOMATIC_STEM_CELL_POPULATION_MAINTENANCE
CELLULAR_OXIDANT_DETOXIFICATION
NEGATIVE_REGULATION_OF_EPITHELIAL_CELL_DIFFERENTIATION
IRON_ION_TRANSMEMBRANE_TRANSPORT
NEGATIVE_REGULATION_OF_VOLTAGE_GATED_CALCIUM_CHANNEL_ACTIVITY
NEGATIVE_REGULATION_OF_RESPONSE_TO_REACTIVE_OXYGEN_SPECIES
SODIUM_ION_HOMEOSTASIS
HYDROGEN_PEROXIDE_METABOLIC_PROCESS
EPITHELIAL_TO_MESENCHYMAL_TRANSITION
RIBOSOME_BIOGENESIS
CELL_CYCLE_CHECKPOINT_SIGNALING
RESPONSE_TO_ESTROGEN
ENTEROENDOCRINE_CELL_DIFFERENTIATION
REGULATION_OF_MAMMARY_GLAND_EPITHELIAL_CELL_PROLIFERATION
POSITIVE_REGULATION_OF_CELL_CYCLE_PROCESS
GLANDULAR_EPITHELIAL_CELL_DIFFERENTIATION
t value
D
Cluster
Patient
PT039
PT058
PT081
PT084
PT089
PT126
Ancestor-like CSC signature
E
KRT19
PTPRC
PDGFRB
F
Gene Ontology Pathways
Ancestor-like CSC signature
gsva score > 0
gsva score < 0
CELLULAR_OXIDANT_DETOXIFICATION
NEGATIVE_REGULATION_OF_STEM_CELL_DIFFERENTIATION
FATTY_ACID_HOMEOSTASIS
EPITHELIAL_TO_MESENCHYMAL_TRANSITION
HYDROGEN_PEROXIDE_METABOLIC_PROCESS
NEGATIVE_REGULATION_OF_CALCIUM_ION_TRANSPORT_INTO_CYTOSOL
IRON_ION_TRANSMEMBRANE_TRANSPORT
CELL_FATE_COMMITMENT
ONE_CARBON_COMPOUND_TRANSPORT
CELL_MATURATION
RETINOL_METABOLIC_PROCESS
CELL_FATE_SPECIFICATION
t value

Supplemental Figure 12, supporting FIG. 8 Expression of ancestor-like CSC signature in tumor tissues. (A) UMAP visualization of scRNA-seq data from all the 11,295 cells in 3 PDX samples (P1, P2, P3), colored by their unsupervised clusters (left), samples (middle) and GSVA score of the ancestor-like CSC signature (right). (B) Expression of marker genes (Seruat, LogNormalized counts) representing cell types. (C) Representative differentially enriched gene ontology (GO) pathways between ancestor-like CSC signature gsva score > 0 and gsva score < 0 in PDX tumor epithelial cells (Figure 8B), using gene set variance analysis (GSVA) (Padj < 0.05). (D) Uniform manifold approximation and projection (UMAP) visualization of scRNA-seq data from all the 1,107 cells in 6 TNBC patients (PT039, PT058, PT081, PT084, PT089, PT126), colored by their unsupervised clusters (left), patients' identifications (middle) and GSVA score of the ancestor-like CSC signature (right). (E) Expression of marker genes (Seruat, LogNormalized counts) representing cell types. (F) Representative differentially enriched gene ontology (GO) pathways between ancestor-like CSC signature gsva score > 0 and gsva score < 0 in tumor epithelial cells showed in (cluster 1 in D), using gene set variance analysis (GSVA) (Padj < 0.05).

Supplementary Table 1

Patient characteristics of breast cancer patients

Patient	Identifier (Patient age)	Histological subtype	ER (IHC)	PR (IHC)	HER2 IHC	KI67 index	Clinical subtype	BRCA1,2 mutations	Clinical drug resistance	stage
P1	K24 (77)	IDC	-	-	-	80%	TNBC	BRCA1, 2	No	IIIC
P2	K53 (84)	IDC	-	-	-		TNBC	BRCA1, 2	No	IIA
P3	No.68 (55)	ILC	-	-	-	50%	TNBC	-	No	IIB
P4	K66 (57)	IDC	-	-	-	17%	TNBC	BRCA2	Yes	IV
P5	K71 (80)	IDC	-	-	-	84%	TNBC	ND	Yes	IV
P6	K61 (70)	ILC	-	ND	-	0.9%	TNBC	ND	No	IIIA

ER, estrogen receptor; PR, progesterone receptor; IHC, immunohistochemistry;
IDC, invasive ductal carcinoma; ILC, invasive lobular carcinoma; ND, not determined. Patients 4 and 5 are relapsed cases after chemotherapy.

Supplementary Table 2

Reagents Used in Organoid Medium

**Reagents**	**Working concentration**
Advanced DMEM/F12	/
Hydrocortisone	1 μM
A83-01	500 nM
FGF7	5 ng/mL
Neuregulin-1	5 nM
EGF	5 ng/mL
SB202190	500 nM
FGF10	20 ng/mL
Y-27632	5 μM
R-spondin3	250 ng/mL
heparin	4 μg/mL
Noggin	100 ng/mL
Primocin	50 μg/mL
N-Acetylcysteine	1.25 mM
Nicotinamid	5 mM
B27	×1
GlutaMax	×1
HEPES (pH7.4)	10 mM
Penicillin-Streptomycin mixed solution	×1

Supplementary Table 3

Patient characteristics of pre- and post-NAC breast cancer patients

Patient	Estrogen receptor (IHC)	Progesterone receptor (IHC)	HER2 IHC	Clinical subtype	Response to NAC	Pathological response to NAC
NP1	-	±	1+	TNBC	PR	Grade 1b
NP2	-	-	1+	TNBC	PR	Grade 1a
NP3	-	-	1+	TNBC	PR	Grade 2a
NP4	-	-	1+	TNBC	PR	Grade 2a
NP5	-	-	1+	TNBC	PR	Grade 1b

PR, partial response.

Supplementary Table 4

Oligonucleotides

**Oligonucleotides**		
Primer *NRP1* Fwd: TACCCTGAGAATGGGTGGAC	this paper	N/A
Primer *NRP1* Rev: CGTGACAAAGCGCAGAAG	this paper	N/A
Primer *IGF1R* Fwd: TTCAGCGCTGCTGATGTG	this paper	N/A
Primer *IGF1R* Rev: AAGTTCCCGGCTCATGGT	this paper	N/A
Primer *FXYD3* Fwd: GGCCAGAAGTCCGGTCA	(Kayed et al., 2006);	N/A
Primer *FXYD3* Rev: AACGGTCCTCCACCCAATTTC	(Kayed et al., 2006);	N/A
Primer *MKI67* Fwd: TGACCCTGATGAGAAAGCTCAA	(Sobecki et al., 2017)	N/A
Primer *MKI67* Rev: CCCTGAGCAACACTGTCTTTT	(Sobecki et al., 2017)	N/A
Primer *ATP1A1* Fwd: TGTTACTGTGGATTGGAGCG	this paper	N/A
Primer *ATP1A1* Rev: CAACCAGTTATGATTACAACGGC	this paper	N/A
Primer *ATP1B1* Fwd: AACCTAAGCCTCCCAAGAATG	this paper	N/A
Primer *ATP1B1* Rev: TGCCCAGTCCAAAATACTCC	this paper	N/A
Primer *GCLC* Fwd: GGCACAAGGACGTTCTCAAGT	(Huang et al., 2017)	N/A
Primer *GCLC* Rev: CAGACAGGACCAACCGGAC	(Huang et al., 2017)	N/A
Primer *ACTB* Fwd: AAGTCCCTTGCCATCCTAAAA	this paper	N/A
Primer *ACTB* Rev: ATGCTATCACCTCCCCTGTG	this paper	N/A
siRNA Negative Control Med GC Duplex #2	Thermo Fisher Scientific	Cat#12935112
siRNA targeting sequence: siFXYD3#1	Thermo Fisher Scientific	Cat# HSS143336
siRNA targeting sequence: siFXYD3#3	Thermo Fisher Scientific	Cat# HSS182369
shRNA targeting sequence: *shATP1B1#1*_5'-CCGGGTGATGAAGTATAACCCAAATCTCGAGATTTGGGTTATACTTCATCACTTTTTG-3'	this paper	N/A
shRNA targeting sequence: *shATP1B1#2* _5'-CCGGGCCGTACAGTTCACCAATCTTCTCGAGAAGATTGGTGAACTGTACGGCTTTTTG-3'	this paper	N/A
